# Edinger-Westphal Urocortin-1 neurons regulate consumption and affect

**DOI:** 10.1016/j.celrep.2025.115814

**Published:** 2025-06-11

**Authors:** Rebecca J. Bluett, Ying Yu, Jordan L. Pauli, Carlos A. Campos, Richard D. Palmiter

**Affiliations:** 1 Department of Biochemistry, University of Washington, Seattle, WA 98195, USA; 2 Howard Hughes Medical Institute, University of Washington, Seattle, WA 98195, USA; 3 Department of Medicine & Diabetes Institute, University of Washington, Seattle, WA 98195, USA; 4 Department of Genome Sciences, University of Washington, Seattle, WA 98195, USA; 5 Lead contact

## Abstract

Affective and consumption circuits in the brain are intertwined, with changes in each domain often affecting the other. Therapeutic interventions that reduce consumption, thereby facilitating weight loss, have tended to increase anxiety and depression. Obesity is a major risk factor for poor health; thus, it is important to understand the circuits and signals that regulate consummatory and affective behavior. Neurons expressing Urocortin-1 (Ucn) in the Edinger-Westphal (EW) nucleus have been hypothesized to play an important role in regulating both consummatory and affective behavioral domains and responses to stress. We generated *Ucn*^*Cre*^ mice to allow measurement of Ucn neuron calcium activity and to directly activate these neurons during consummatory behaviors and repeated restraint stress. Our findings provide evidence for Ucn neuron regulation of consummatory behaviors and some affective behaviors.

## In brief

Bluett et al. generate a *Ucn*^*Cre*^ mouse line, showing that Edinger-Westphal Urocortin-1 neuron activity exhibits a biphasic pattern during hedonic consumption and increases during active coping. The activation of Urocortin neurons reduces consumption but minimally impacts affective-like behavior, suggesting these neurons could be useful targets in the treatment of obesity.

## Graphical Abstract

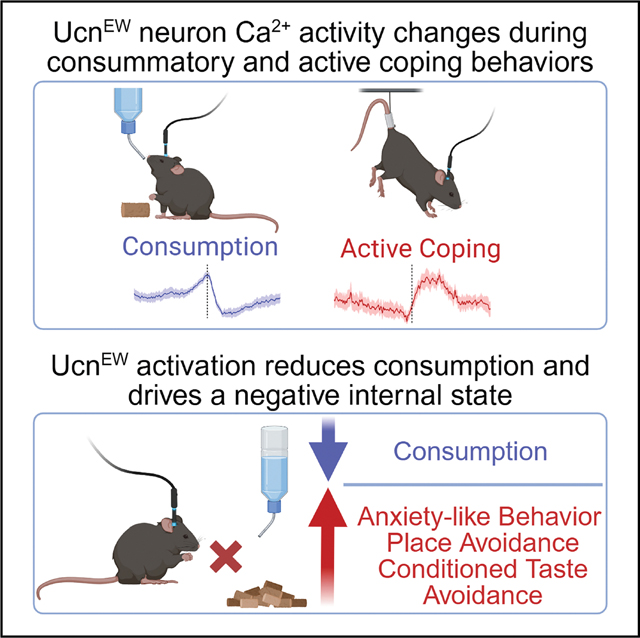

## INTRODUCTION

Caloric deficit, pleasure seeking, and stress coping can drive consumption. While numerous physiological processes interact to produce obesity, elevated consumption for pleasure (hedonic need) regardless of hunger state (homeostatic need) is associated with obesity,^1^ which is a major driver of poor health.^2,3^ Delineating the brain circuits that regulate these consummatory behaviors may reveal therapeutic approaches to reduce obesity and adiposity-related negative health consequences.^4^ Centrally projecting Edinger-Westphal (EW) nucleus neurons are hypothesized to regulate consumption, particularly in the context of stress.^5–9^ EW lesion decreases consumption, and EW neurons express receptors for the feeding-related peptides leptin, ghrelin, and melanocortin.^10–14^ EW neurons have also been implicated in regulating alcohol intake and may bridge reward and stress systems.^14–18^ Many EW neurons express the Urocortin-1 (Ucn) peptide, which is closely related to corticotropin-releasing hormone (CRH), which regulates stress responses.^7,8,19–21^ Stress and affective states strongly impinge on consummatory behaviors (and vice versa).^22^ Pharmacological interventions that reduce adiposity tend to exhibit negative psychological impacts, such as increasing anxiety, depression, and suicidal ideation.^23,24^ Therefore, in seeking consumption-regulating therapeutics, it is critical to assess anxiety- and depressive-like behaviors. Stress increases Fos expression, a proxy for neuronal activation, in Ucn-expressing EW neurons (Ucn^EW^),^25–31^ although not all studies have replicated this.^32^ Likewise, the impact of EW lesions on stress and affective-like behaviors has been mixed.^10,33^ Work targeting chimeric antigen receptor T (CART)-expressing (largely Ucn-overlapping) EW neurons has shown that these neurons can drive arousal, maternal nesting, and anxiety-like behaviors, but potential roles in regulating consummatory behaviors and stress responses require further testing.^34–36^

To test if Ucn^EW^ neurons regulate consumption, we developed a mouse line with Cre recombinase targeted to the *Ucn* locus and used genetic crosses to elicit Ucn-specific expression of GCaMP or channelrhodopsin 2 (ChR2). Fiber photometry showed a complex relationship between Ucn^EW^ calcium activity and consummatory behavior, with hedonic consumption eliciting a pre-consumption increase followed by a substantial consumption-related decrease in activity. Optogenetic activation of Ucn^EW^ neurons reduced food and fluid consumption in multiple contexts. While Ucn^EW^ activity was strongly increased during active coping, exogenous activation had an inconsistent effect on coping and anxiety-like behaviors. These results support the possibility that Ucn^EW^ neurons and associated circuits could be of interest in the search for therapeutic targets to reduce consumption, although it will be important to carefully test for negative psychiatric side effects.

## RESULTS

### Characterization of a gene-targeted Ucn^Cre^ line of mice

We generated a mouse line with Cre recombinase targeted to the *Ucn* locus by inserting an IRES-Cre:GFP cassette just beyond the termination codon. We crossed this *Ucn*^*Cre*^ to the *Rosa26*^*LSL-tdTomato*^ (tdT) reporter line. Offspring of the cross exhibited tdT expression in a narrow swath of midline cells spanning bregma levels ~2.8–4.0, matching previous reports of *Ucn* expression^35–38^ (Figures S1A–S1D; full-brain tdT immunostaining, Zenodo: https://doi.org/10.5281/zenodo.11492903). We did not observe tdT expression in other regions previously shown to express Ucn, such as the lateral superior olive.^20,39^ Using fluorescence *in situ* hybridization (FISH), we verified expression fidelity, with 86% of cells co-expressing *Ucn* and tdT (Figures S1E and S1F). FISH showed overwhelming *Ucn* and *Cck* co-expression (95%) and partial *Ucn* and *Adcyap1* overlap (52%) but minimal co-expression of *Ucn* and *Slc17a6* (7%) or *Tac1* (1%) (Figures S1G–S1I). These co-expression results agree with previous reports and further validate that Cre was appropriately targeted.^35,36^ We also observed tdT expression bilaterally in the anterior insular cortex (Figures S1J and S1K) but were unable to confirm Ucn or Cre expression in the adult brain via FISH or delivery of viral vectors with Cre-dependent reporter expression, suggesting transient developmental *Ucn* gene expression in this population. Stereotaxic delivery of AAV1-hSyn-DIO-YFP and AAV1-Ef1a-DIO-synaptophysin:mCherry viral vectors to the EW of *Ucn*^*Cre*^ mice elicited expression of YFP and mCherry in EW cells and many putative projection targets, including the periaqueductal gray, multiple hypothalamic areas, the bed nucleus of the stria terminalis, medial septum, medial vestibular nucleus, and interpeduncular nucleus (Figures S1M–S1R).^36,38,40,41^ Some projection regions, e.g., the striatum, exhibited a substantial mismatch between expression from viral vectors and expression in the *Ucn*^*Cre*^::tdT cross (Figures S1S versus S1T; Zenodo data: https://doi.org/10.5281/zenodo.11492903 comparing tdT and synaptophysin images 13–17). Several factors could result in these differences, including additional tdT expression from cortical neuron projections, incomplete viral transfection, or tissue damage from viral expression.

### Ucn^EW^ neuron activity pattern differs during hedonic and homeostatic consumption

To elicit the expression of a fluorescent calcium indicator in Ucn^EW^ neurons, we crossed *Ucn*^*Cre*^ to a line with Cre-dependent expression of GCaMP6s (Ai162). We placed fiber-optic cannulas over the EW and used fiber photometry to record Ucn^EW^ calcium (Ca^2+^) activity (Figures S2A–S2C).

**Figure 1.**
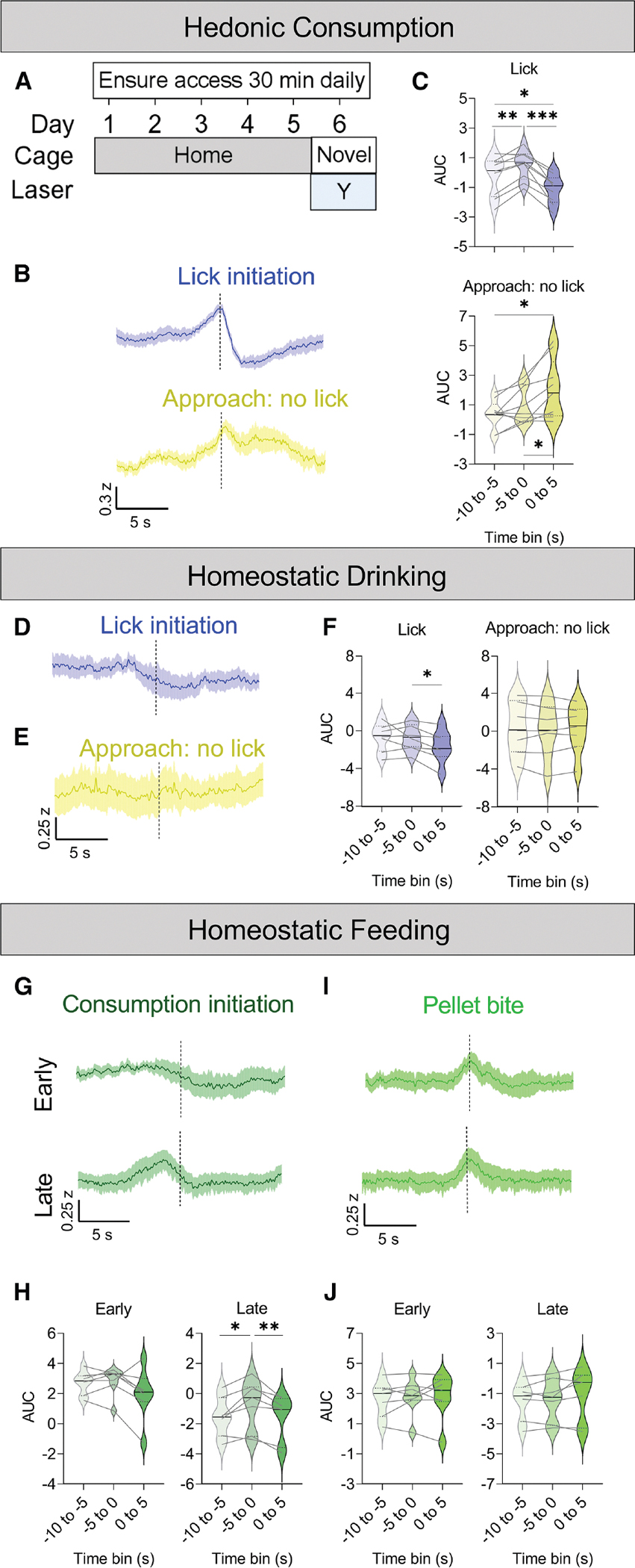
Ucn^EW^ neuron activity pattern differs during hedonic and homeostatic consumption (A) Novelty-induced hypophagia (NIH) schematic. (B) Fiber photometry *Z* score, peri-event plots of 10 s before and after (top) lick bout initiation for familiar palatable drink (Ensure) and (bottom) sipper approaches without lick in a novel, empty brightly lit cage on day 6 of Ensure exposure. Averaged from *n* = 9 *Ucn*^*Cre*/+^::Ai162^+/−^ mice. (C) Area under the curve of *Z* scores in (B) for lick (top) and approach without lick (bottom). (D) Fiber photometry *Z* score, peri-event plots of 10 s before and after lick initiation in the home cage under dim light after overnight water deprivation. Averaged from *n* = 7 *Ucn*^*Cre*/+^::Ai162^+/−^ mice. (E) Fiber photometry *Z* score, peri-event plots of 10 s before and after water sipper approaches without lick initiation after overnight water deprivation. Averaged from *n* = 7 mice. (F) Area under the curve of *Z* scores for (D) on the left and for (E) on the right. (G) Fiber photometry *Z* score, peri-event plots of 10 s before and after consumption bout initiation from 0 to 15 min (top, “early”) and from 15 to 30 min (bottom, “late”) of home-cage refeeding under dim light after an overnight fast. Averaged from *n* = 7 *Ucn*^*Cre*/+^::Ai162^+/−^ mice. (H) Area under the curve of *Z* scores in (G). (I) Fiber photometry *Z* score, peri-event plots of 10 s before and after pellet bite initiation from 0 to 15 min (top, “early”) and from 15 to 30 min (bottom, “late”) of refeeding after an overnight fast. Averaged from *n* = 7 mice. (J) Area under the curve of *Z* scores in (I). (B, D, G, and I) Data are shown as mean ± SEM. (C, F, H, and J) Data are shown as median ± quartiles. Repeated measures one-way ANOVA with Tukey test for multiple comparisons. See Table S1 for more experimental and statistical details. *p < 0.05, **p < 0.01, ***p < 0.001.

In sated animals, Ucn^EW^ neurons responded biphasically to the hedonic consumption of a familiar, palatable drink (Ensure) in the novelty-induced hypophagia (NIH) novel-cage test. Calcium activity significantly increased in the 5 s before licking and then decreased below baseline in the 5 s following lick initiation (Figures 1A–1C). In contrast, when mice approached the sipper but did not lick, Ucn^EW^ activity was elevated during the 5 s after the approach (Figures 1B and 1C). To determine if Ca^2+^ activity simply reflected locomotion (which increases during sipper approach and decreases during licking), we compared mouse velocity to Ca^2+^ activity during licking and grooming bouts in this test. Calcium activity decreased after lick onset but increased after grooming onset, while velocity decreased in both cases (Figures S2D–S2F). During home-cage Ensure consumption, Ca^2+^ activity significantly decreased after lick onset, although here, the small increase in activity before lick onset was not significant (Figure S2G).

To test if Ucn^EW^ neurons respond similarly to homeostatic consumption, we measured Ca^2+^ activity in the home cage after overnight water or food deprivation. During a 30-min rehydration period, Ucn^EW^ activity remained unchanged before licking but significantly decreased during the 5 s after water consumption was initiated (Figures 1D and 1F, left) with no change in activity when mice approached the sipper without licking (Figures 1E and 1F, right). Activity was also unchanged during rearing (during which body position changes similarly to licking bouts) in other parts of the cage (Figure S2H). Consumption did not significantly affect Ucn^EW^ activity during the first half of a 30-min home-cage refeeding session after an overnight fast (Figures 1G and 1H, “early”). However, during the second half of the test, Ca^2+^ activity significantly increased during the 5 s before consumption and returned to baseline by 5 s after initiating consumption (Figures 1G and 1H, “late”). Consumption bouts (periods of chewing) were often preceded by biting the large chow pellet to remove a smaller chunk to hold and consume. The Ca^2+^ transients appeared to temporally correspond more closely to the onset of pellet biting than chewing, but pellet-biting-aligned activity did not reach statistical significance (Figures 1I and 1J). Activity was unchanged during grooming in the refeed test (Figure S2I), in contrast to the grooming-related activity during NIH (Figure S2F), which may reflect a functional difference in grooming in a novel, anxiogenic environment versus in the home cage.

These data show that hedonic and homeostatic fluid and food consumption alter Ucn^EW^ neuron activity, although the response profile is complex, with anticipatory increases in some contexts (sated Ensure and hungry chow) and decreases during consumption in other contexts (sated Ensure and thirsty water).

### Ucn^EW^ neuron activation reduces consumption

We hypothesized that increasing Ucn^EW^ activity would influence consumption. To test this, we crossed Ucn^EW^ to the *Rosa26*^*LSL-ChR2*^ line of mice (Ai32) to drive the expression of ChR2-EYFP in Ucn neurons. Fiber-optic cannulas were targeted to EW to enable laser-mediated Ucn^EW^ stimulation (Figures S3A–S3D).

**Figure 2.**
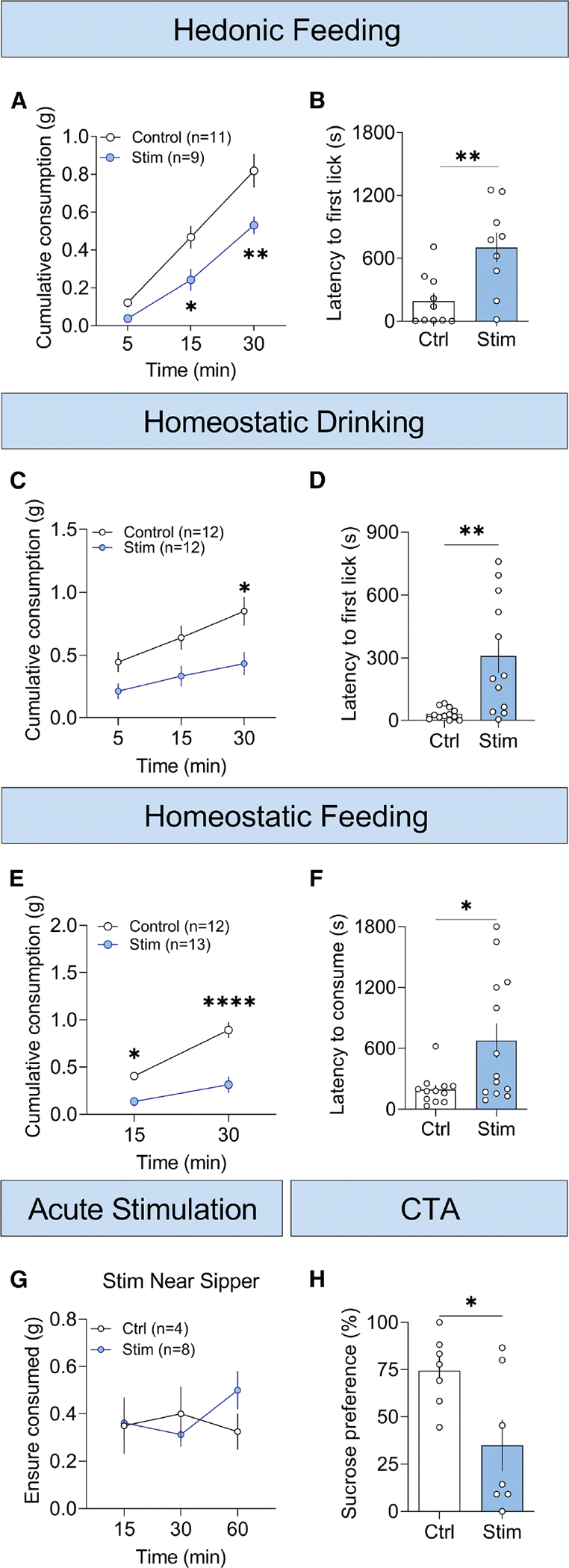
Activation of Ucn^EW^ neurons reduces consumption (A) Cumulative Ensure (familiar, palatable drink) consumption in the NIH novel-cage test (new, brightly lit, empty cage). Controls are *Ucn*^+/+^::Ai32^+/−^ and Stim (photostimulation) are *Ucn*^*Cre*/+^::Ai32^+/−^. (B) Latency to first lick in the NIH novel-cage test. (C) Cumulative water consumption during 30-min home-cage rehydration period after overnight water deprivation. Controls are *Ucn*^+/+^::Ai32^+/−^ and Stim are *Ucn*^*Cre*/+^::Ai32^+/−^. (D) Latency to first lick after overnight water deprivation. (E) Cumulative chow consumption during 0-to-15- and 0-to-30-min bins of the home-cage refeeding period after overnight fasting. Controls are *Ucn*^+/+^:: Ai32^+/−^ and Stim are *Ucn*^*Cre*/+^::Ai32^+/−^. (F) Latency to first bite after an overnight fast. (G) Ensure (familiar, palatable drink) consumption time course with optogenetic stimulation triggered by sipper proximity in a novel, brightly lit, empty cage. Controls are *Ucn*^+/+^::Ai32^+/−^ and Stim are *Ucn*^*Cre*/+^::Ai32^+/−^. (H) Sucrose preference in the 2-bottle conditioned taste avoidance (CTA) test where mice had access to water and 5% sucrose. CTA was tested after 3 conditioning days of 30-min sucrose access followed by 30-min Ucn^EW^ photostimulation. See Figure S3I for CTA schematic. Controls are *Ucn*^+/+^::Ai32^+/−^ and Stim are *Ucn*^*Cre*/+^::Ai32^+/−^. (A–H) Data are shown as mean ± SEM. (A, C, E, and G) Repeated measures two-way ANOVA with Bonferroni multiple comparison test. (B, D, F, and H) Two-tailed unpaired t test with Welch’s correction. *p < 0.05, **p < 0.01, ***p < 0.001, ****p < 0.0001.

Photostimulation of Ucn^EW^ neurons (20 Hz, 2 s on, 2 s off; 5–10 mW) significantly reduced consumption and increased latency to first lick in the NIH novel-cage test (Figures 2A and 2B). Other animals were photostimulated during the first home-cage Ensure exposure; this significantly increased latency to first lick and decreased consumption (Figure S3E). Consumption remained lower throughout several days of Ensure access without additional neuronal stimulation (Figure S3E, right). When a novel, high-fat food pellet was introduced in the home cage, Ucn^EW^ activation similarly reduced consumption (Figure S3F). Additionally, when mice were given Ensure in the home cage after completing the standard NIH paradigm, Ucn^EW^ stimulation significantly reduced Ensure consumption, although to a lesser degree than in the anxiogenic conditions of the NIH novel-cage and food neophobia tests (Figure S3G).

ChR2-mediated Ucn^EW^ stimulation reduced homeostatic water consumption after overnight water deprivation and significantly increased latency to first lick (Figures 2C and 2D). Similarly, Ucn^EW^ stimulation reduced homeostatic chow consumption and increased latency to first bite after overnight fasting (Figures 2E and 2F). Stimulation in this context also reduced the distance traveled but with a later onset than the anorexic effect (Figure S3H). There was no correlation between consumption and distance traveled, indicating that reduced consumption was not dependent on reduced locomotion (Figure S3H). Overall, prolonged activation of Ucn^EW^ neurons substantially decreased or delayed consumption in every context.

To determine if stimulation had an immediate, time-locked effect on consumption, we repeated the NIH novel-cage test with optogenetic stimulation triggered by sipper proximity and found that consumption was unchanged (Figure 2G). Thus, ongoing activity of these neurons is required to reduce consumption. When Ucn^EW^ neurons were stimulated for 30 min following 30-min access to a novel, palatable drink (5% sucrose), mice reduced subsequent ingestion of that drink without changing water intake relative to controls (Figures 2H and S3I). This conditioned taste avoidance (CTA) suggested that Ucn^EW^ activation induces an aversive internal state, which may be part of the mechanism by which activation reduces consumption.

### Ucn^EW^ neuron activity increases during active coping

**Figure 3.**
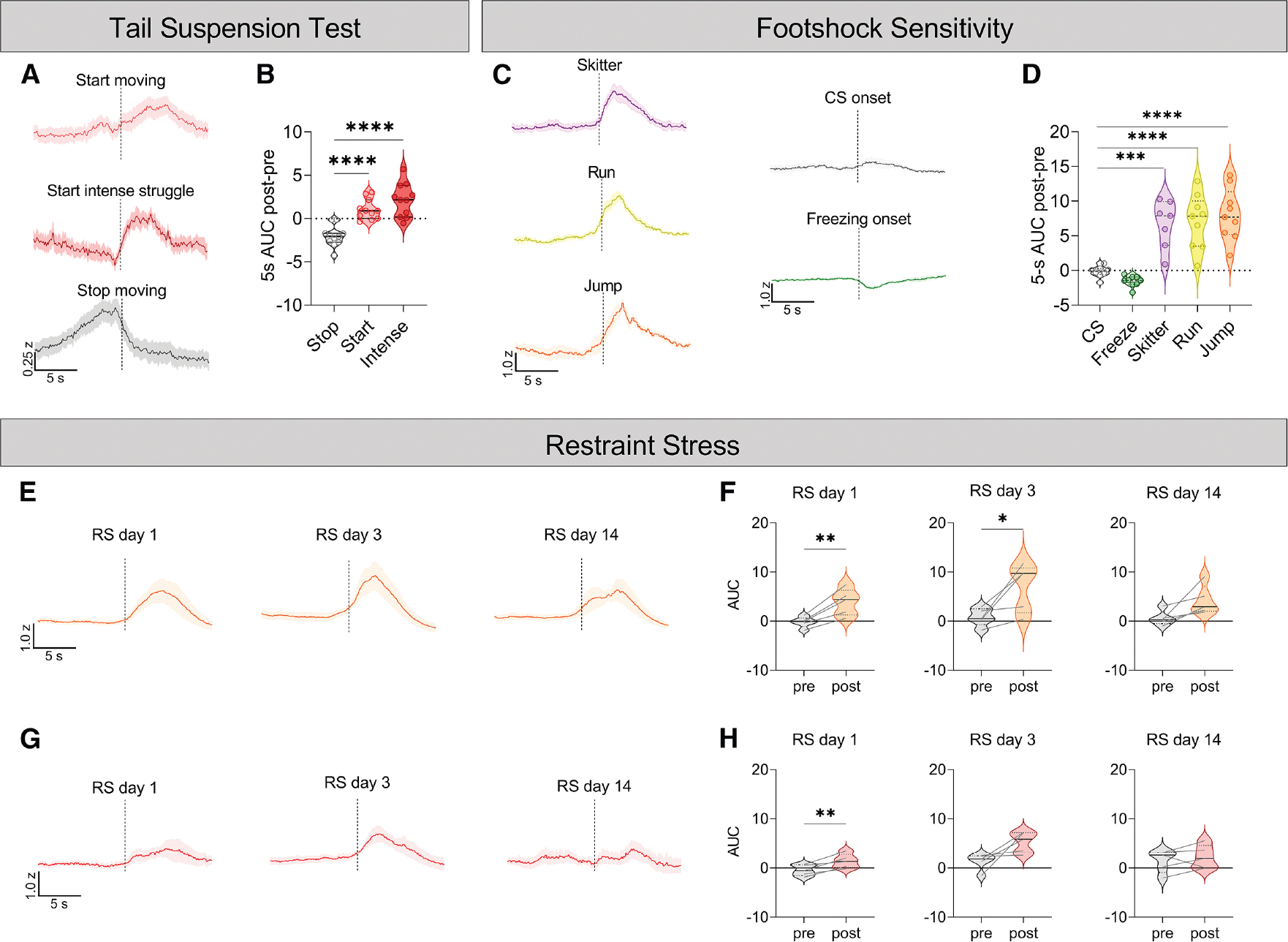
Ucn^EW^ neuron activity increases during active coping (A) Fiber photometry *Z* score, peri-event plots of 10 s before and after mice stop moving (top), start small movements (middle), and start intense struggling bouts (bottom) in a 6-min tail suspension test, during which mice hung from their tails >40 cm above the ground. Averaged from *n* = 11 *Ucn*^*Cre*/+^::Ai162^+/−^ mice. (B) Area under the curve for the 0-to-5-s bin minus the −5-to-0-s bin of *Z* scores in (A). (C) Fiber photometry *Z* score, peri-event plots of (left) 10 s before and after onset of a 2-s foot shock (intensity: 0.07–0.4 mA) with bouts grouped by behavioral responses: (top) small, skittering movements, (middle) running, and (bottom) jumping. (Right) Fiber photometry *Z* score, peri-event plots of 10 s before and after (top) initiation of the audiovisual conditioned stimulus (CS) and (bottom) freezing. Averaged from *n* = 7–9 *Ucn*^*Cre*/+^::Ai162^+/−^ mice (not all animals performed all behaviors). (D) Area under the curve for the 0-to-5-s bin minus the −5-to-0-s bin of *Z* scores in (C). (E) Fiber photometry *Z* score, peri-event plots of 10 s before and after initiation of intense struggling during the 30-min restraint on (left) restraint stress day 1 (RS1), (middle) RS3, and (right) RS14. Restraint stress took place in the first 4 h of the light cycle. Averaged from *n* = 5 *Ucn*^*Cre*/+^::Ai162^+/−^ mice. (F) Area under the curve of *Z* scores in (E), with “pre” indicating time bin −10 to −5 s and “post” indicating time bin 0 to 5 s. (G) Fiber photometry *Z* score, peri-event plots of 10 s before and after initiation of small mobility bouts on (left) RS1, (middle) RS3, and (right) RS14. Averaged from *n* = 5 *Ucn*^*Cre*/+^::Ai162^+/−^ mice. (H) Area under the curve of *Z* scores in (G), with “pre” indicating time bin −10 to −5 s and “post” indicating time bin 0 to 5 s. (A, C, E, and G) Data are shown as mean ± SEM. (B, D, F, and H) Data are shown as median ± quartiles. (B) Ordinary one-way ANOVA with Tukey test for multiple comparisons. (D) Brown-Forsythe ANOVA with Dunnett’s multiple comparisons. (F and H) Two-tailed paired t test. *p < 0.05, **p < 0.01, ***p < 0.001, ****p < 0.0001.

We next tested if Ucn^EW^ activity changes during stressful experiences. Consistent with a recent report,^36^ Ucn^EW^ neurons were strongly activated by a 10-s tail suspension off the cage bottom, during which mice struggled (Figure S4A). Fiber photometry recording during a 6-min tail suspension test including bouts of struggling and immobility revealed that Ucn^EW^ Ca^2+^ activity increased at the start of small movement bouts and bouts of intense whole-body struggling and returned to baseline when movement stopped (Figures 3A and 3B). Activity was unchanged when scruffed mice were held aloft (∼45 cm) and rotated from belly up to side to belly down, suggesting that activity during tail suspension was unlikely to be driven by vestibular changes related to changing body position (Figure S4B). These data suggest that Ucn^EW^ neurons are active during periods of active coping.

Foot-shock sensitivity testing confirmed, as others have shown, that shock drives Ucn^EW^ activity.^36,42^ While shock increased Ca^2+^ activity, the increase did not scale with behavioral response intensity, with similar activation across a range of escape behaviors, from minor skittering movements to running and jumping (Figures 3C and 3D). Similarly, Ca^2+^ activity did not substantially differ across shock intensities from 0.07 to 0.4 mA (Figure S4C), suggesting the response is not directly related to stimulus intensity. There was also no difference in Ca^2+^ response between cued and uncued shocks (Figure S4D). The onset of the audiovisual cue did not alter Ca^2+^ activity, but activity was significantly reduced at the onset of freezing (Figures 3C, 3D, and S4E). Freezing is a passive coping response, while escape behaviors during shock and struggling during tail suspension are active coping responses.^43–45^ Thus, Ucn^EW^ activity increases during active coping and decreases during passive coping in some contexts.

Next, we tested if Ucn^EW^ activity during active coping changes with repeated exposures to the same stressor. Calcium activity significantly increased in the 5 s after the onset of intense struggling bouts on restraint stress day 1 (RS1) and RS3 but not RS14 (Figures 3E and 3F). Additionally, Ca^2+^ activity significantly increased at the onset of smaller mobility bouts on RS1 but not RS3 or RS14 (Figures 3G and 3H). These data show that Ucn^EW^ activity habituates across repeated exposure to restraint stress.

### Activation of Ucn^EW^ neurons is aversive

**Figure 4.**
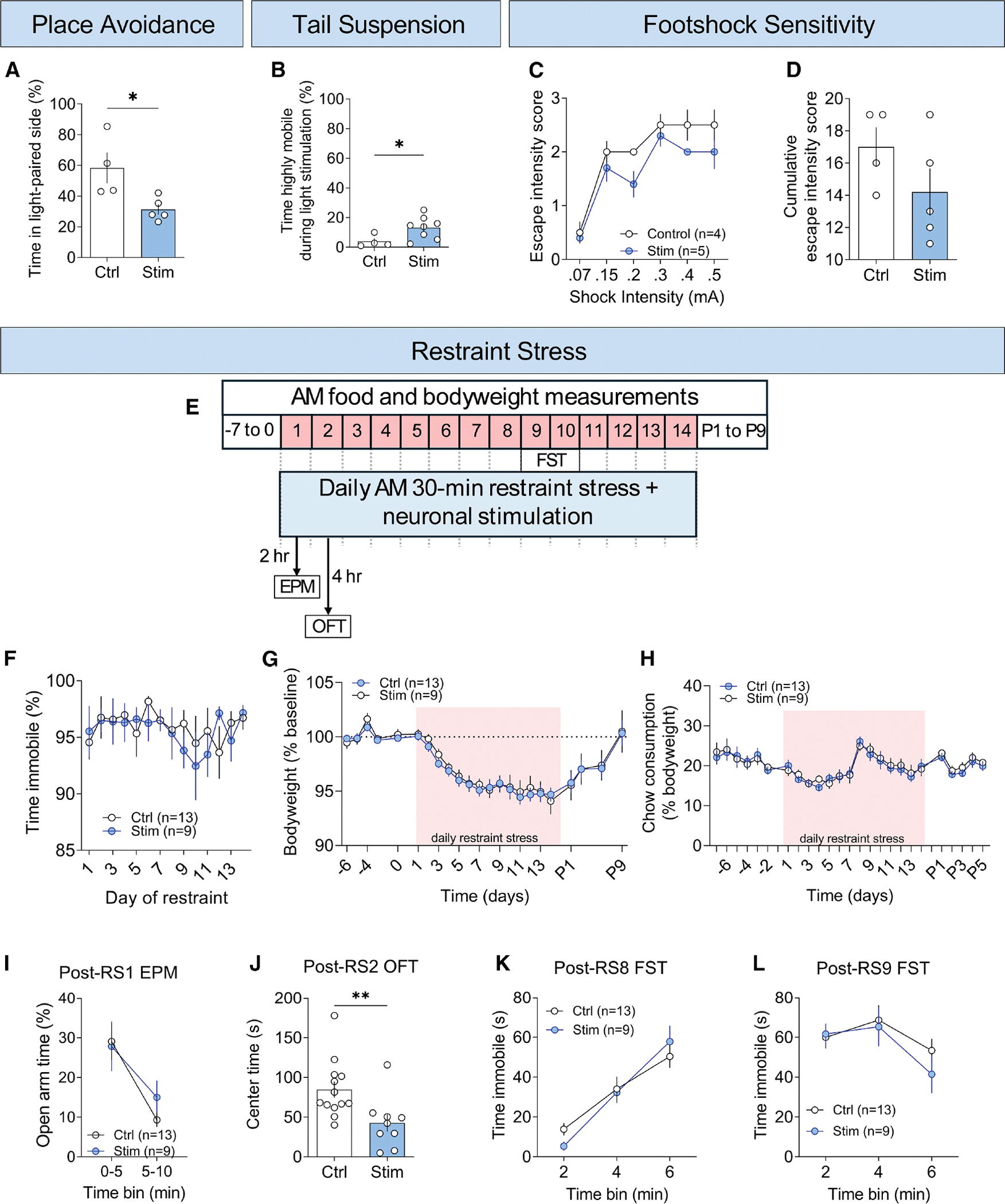
Activation of Ucn^EW^ neurons is aversive (A) Percentage of time spent in the light-paired side during the 20-min light-active phase of real-time place avoidance testing in a 2-sided arena with distinct wall patterns and floor textures. Controls are *Ucn*^+/+^::Ai32^+/^ and Stim are *Ucn*^*Cre*/+^::Ai32^+/−^. (B) Total time spent highly mobile during tail suspension test, during which mice hung from their tails >40 cm above the ground, with photostimulation (2–4 and 6–8 min). Controls are *Ucn*^+/+^::Ai32^+/−^ and Stim are *Ucn*^*Cre*/+^::Ai32^+/−^. (C) Average escape intensity score for each foot-shock intensity during the foot-shock sensitivity test, during which mice received 2-s foot shocks at the end of an audiovisual cue (no response = 0, skitter = 1, run = 2, and jump = 3). Controls are *Ucn*^+/+^::Ai32^+/−^ and Stim are *Ucn*^*Cre*/+^::Ai32^+/−^. (D) Average cumulative escape intensity score across foot-shock intensities. (E) Schematic of repeated restraint stress. (F) Immobility during 30-min restraint stress (RS) across 14 consecutive daily RS exposures. Controls are *Ucn*^+/+^::Ai32^+/−^ and Stim are *Ucn*^*Cre*/+^::Ai32^+/−^. (G) Body weight during repeated RS. Negative numbers indicate pre-stress days and P# indicates days post-stress. The average of days −6 to 1 was used as a baseline to calculate body weight as a percentage of the baseline. (H) Chow consumption during repeated RS. Consumption normalized to body weight to control for size differences. (I) Percentage of time spent in open arms of the elevated plus maze (EPM) test conducted ∼2 h after RS1. (J) Center time in the first 10 min of a 30-min open-field test (OFT) conducted ∼4 h after RS2. (K) Time immobile in the forced swim test (FST) conducted ∼22 h after RS8. (L) Time immobile in FST conducted ∼22 h after RS9. (A–D and F–L) Data are shown as mean ± SEM. (A, B, D, and J) Two-tailed unpaired t test. (C, F–I, K, and L) Repeated measures two-way ANOVA with Bonferroni test for multiple comparisons. *p < 0.05, **p < 0.01.

The large changes in Ca^2+^ activity during active coping bouts supported the hypothesis that activation of these neurons drives a negative internal state, suggesting that exogenous neuronal activation would be aversive. Indeed, Ucn^EW^ stimulation in naive animals produced real-time place avoidance, significantly decreasing the percentage of time mice spent in the stimulation-paired side of a two-sided chamber (Figures 4A and S5A). Ucn^EW^ photostimulation slightly increased the percentage of time mice were highly mobile in the tail suspension test, although overall immobility was not significantly affected (Figures 4B and S5B). Foot-shock responsivity, as measured by the average escape intensity score (no response = 0, skitter = 1, run = 2, and jump = 3), was not significantly altered by Ucn^EW^ stimulation (Figures 4C and 4D).

We also tested if Ucn^EW^ photostimulation during repeated restraint stress would change struggling or affective behaviors (Figure 4E). The percentage of time spent immobile during 14 daily 30-min restraint-stress exposures was unchanged by Ucn^EW^ stimulation (Figure 4F). We wondered if stimulation altered the stress experience in a way that might modulate other stress-related outcomes. For example, it has been hypothesized that Ucn^EW^ neurons regulate stress-related changes in energy homeostasis.^5,6^ To test this, we measured body weight, food intake, and post-stress anxiety-like and depressive-like behaviors. Stress-induced changes in body weight and food intake were not significantly impacted by Ucn^EW^ activation during restraint stress (Figures 4G and 4H). Likewise, stimulation during RS1 did not impact post-stress, anxiety-like behavior in the elevated plus maze (EPM), as measured by the percentage of open-arm time (2 h after RS1; Figure 4I). In unstressed mice, Ucn^EW^ stimulation was anxiogenic in EPM but not in open-field testing (OFT) (Figure S5C). After photostimulation during RS2, mice exhibited a mild anxiety-like phenotype with decreased center time in the first 10 min of 30-min OFT performed 4 h after stress (Figure 4J). Distance traveled in these post-stress tests was unchanged (Figure S5D). Time immobile, a measure of depressive-like behavior in the forced swim test (FST; performed 23 h after RS8 and RS9), was unaffected by photostimulation during RS (Figures 4K and 4L).

These data demonstrate that Ucn^EW^ stimulation is mildly aversive and anxiogenic in some contexts but do not support the hypothesis that Ucn^EW^ neurons regulate stress-induced changes in energy homeostasis or depressive-like states.

## DISCUSSION

Despite a large body of research that implicates interdependent, stress-sensitive circuits in the regulation of consummatory and affective behaviors, many neural populations that regulate these behaviors remain untested.^22,46–49^ There has been much speculation that EW peptidergic neurons, specifically Ucn^EW^ neurons, are involved in energy homeostasis and affective behavior, particularly in the context of stress.^5,6,8^ While EW peptidergic neurons have been shown to regulate arousal, maternal nesting, and some anxiety-like behaviors, evidence remains mixed regarding their role in consummatory, affective, and stress-related behaviors. To fill this gap, we generated a *Ucn*^*Cre*^ mouse line and performed genetic crosses to Cre-dependent calcium indicator and optogenetic effector lines to measure calcium signaling and drive neuronal activity in Ucn^EW^ neurons.

Our data establish a role for Ucn^EW^ neurons in regulating energy-deficit-driven (homeostatic) and palatability-driven (hedonic) consumption, although this may be secondary to the induction of a negative internal state. These data contrast with one previous study that reported no change in the Ca^2+^ activity of peptidergic EW neurons during spontaneous home-cage feeding.^35^ Here, we show that Ucn^EW^ neurons exhibit a complex pattern of Ca^2+^ activity across different consumption contexts during Ensure intake by sated mice and chow consumption by hungry mice (in the second half of the refeed). This increase could reflect anticipation of caloric intake, which would explain its absence during thirsty water consumption. However, it is least apparent at the beginning of a refeeding period when mice are hungriest and most pronounced in NIH where sated mice have access to a palatable drink, suggesting, rather, that it may be related to expected hedonic value. With sated Ensure and thirsty water consumption, but not hungry chow consumption, Ca^2+^ activity decreases after consumption initiation, which is, again, more pronounced in the hedonic context. This may, however, be a limitation of our freely moving refeed paradigm, where consumption is composed of multiple phases (approach, sniff, handle, bite, and ingest). Future studies using head-fixed behavior may be able to map activity to a specific phase of consumption. Overall, a bias toward the regulation of hedonic consumption supports the idea that Ucn^EW^ signaling impinges on reward behavior, which could have implications for obesity and binge-eating disorders or, as others have suggested, alcohol and substance use.^15,30,50^ Future studies could provide clarity on this possibility by using head-fixed recording to compare bout-by-bout Ucn^EW^ activity while manipulating homeostatic need state and palatability.

Ucn^EW^ activation increased consumption latency across homeostatic and hedonic conditions and reduced overall consumption. Previous studies demonstrated that Ucn peptide infusion into numerous putative projection targets of Ucn^EW^ neurons potently reduced consumption.^51–57^ Low doses of centrally administered Ucn reduced food intake without conditioning taste avoidance, while higher doses produced CTA.^51,58^ Our stimulation of Ucn^EW^ neurons produced both a moderate CTA and real-time place avoidance, suggesting induction of an aversive internal state that could result in reduced consumption. Future studies measuring stimulationinduced peptide release in downstream brain regions are necessary to determine if these effects are Ucn mediated. These data complicate the interpretation of Ucn^EW^ activation reducing consumption, as aversive internal states often indirectly reduce consummatory behaviors. However, central Ucn administration can impact metabolism, and EW peptidergic neurons have been shown to respond to energy homeostasis signals like leptin, ghrelin, and melanocortin, suggesting a direct role in energy homeostasis is possible.^11–13,52^ Future studies using less intense stimulation or targeting distinct projections may be able to separate decreased consumption from an aversive internal state.

Ucn^EW^ calcium activity robustly increased during active coping bouts. These active coping bouts all involve abrupt changes in motor behavior, thus generally agreeing with a recent report of increased EW neuron activity during acute restraint, tail suspension, and electric shock, as well as during anesthesia and ethanol-induced uncoordinated locomotion.^36^ However, those authors attributed this activity to a “loss of motor control,” while our data show that activity is specific to short struggling bouts and not maintained throughout a longer period of tail suspension or restraint, suggesting that activity is related to the active behavioral response rather than to a loss of control per se. Interestingly, we found that the calcium activity increase during active coping bouts decreased by RS14, suggesting that EW neurons habituate to repeated stress, contrary to previous reports.^29,59^

We hypothesized that optogenetic Ucn^EW^ stimulation during restraint stress would enhance the active coping-related activity we saw with fiber photometry and drive higher levels of active coping, especially at the 14-day time point. Surprisingly, maintaining higher levels of Ucn^EW^ activity throughout the restraint-stress paradigm did not impact active coping behaviors. Further, activation during restraint stress had little effect on post-stress anxiety-like behavior and no effect on depressive-like behavior, chow intake, or body weight. However, others have shown increases in Fos and Ucn expression in Ucn^EW^ neurons at 4–24 h after stress,^25,26,28,42,60^ so future studies should test the possibility that Ucn^EW^ stimulation during the post-stress period could impact stress responses. Altogether, our data directly contradict the prominent hypotheses that Ucn^EW^ neurons regulate stress habituation and stress-related energy homeostasis^5,6,13^ and suggest that this circuit may be a candidate for consumption-inhibiting therapeutics.

### Limitations of the study

We showed that Ucn^EW^ neurons exhibit a biphasic calcium response during hedonic feeding, which is only partially recapitulated during homeostatic drinking or feeding. However, we could not distinguish between a biphasic response in the same neurons and distinct subpopulation response profiles. Unfortunately, efforts to implant miniscopes to record single-cell calcium activity resulted in high post-surgical mortality rates, possibly due to occlusion of the cerebral aqueduct. We further showed that exogenous activation of Ucn^EW^ neurons disrupted hedonic and homeostatic consumption in our freely moving behavioral paradigms, but future studies using head-fixed behavioral paradigms would allow more precise mapping of activity and regulatory capacity across different need states and hedonic values. Photostimulation also produced a mildly aversive internal state, which could be the mechanism by which consumption is reduced. Our experiments were not able to separate these outcomes, but future work manipulating Ucn^EW^ neuron projections or peptides may be able to. We also did not perform loss-of-function experiments to test the necessity of Ucn^EW^ neuron activity in consummatory behavior. While we included both male and female mice in our studies, we were not powered to detect sex differences.

### RESOURCE AVAILABILITY

#### Lead contact

Requests for further information and resources will be fulfilled by the lead contact, Richard D. Palmiter (palmiter@uw.edu).

#### Materials availability

The *Ucn*^*Cre*^ mouse line generated in this study has been deposited at Jackson Laboratories (Stock #39757).

#### Data and code availability

- Data will be made available upon reasonable request to the lead contact.
- All original code generated in the fiber photometry analysis of this study has been deposited online at Zenodo: https://doi.org/10.5281/zenodo.11624342 and is available online as of the date of publication.
- Additional images from immunostaining experiments can be found at Zenodo: https://doi.org/10.5281/zenodo.11492903.
- Any additional information required to reanalyze the data reported in this work paper is available from the lead contact upon request.

## STAR★METHODS

Detailed methods are provided in the online version of this paper and include the following:

### EXPERIMENTAL MODEL AND STUDY PARTICIPANT DETAILS

All experiments were approved by the Institutional Animals Care and Use Committee at the University of Washington. Animals were group-housed with littermates on a 12-h light cycle at ∼22°C with food and water available *ad libitum*, except where behavioral experiments required single-housing. Experiments were performed during the light cycle in dimly lit rooms except where noted in specific behavioral paradigms. Three lines of mice with Cre-dependent (lox-stop-lox, LSL) effector genes were used in these studies; *Rosa26*^*LSL-tdTomato*^ (Ai14, Jackson Lab stock #07914), *Rosa26*^*LSL-ChR2*^ (Ai32, Jackson Lab stock #24109) and a Cre-dependent GCaMP6 line (Ai162, Jackson lab stock #31562). Adult (>8 week old) male and female mice from the same litters were used. Each behavioral cohort generally experienced 3–4 experimental paradigms beginning with the least stressful and ending with the most stressful. The experiments were not powered to detect significant differences related to sex, but none were noted. All mice were on a C57BL/6J genetic background. For experiments reported here, the mice mentioned above were bred with the *Ucn*^*Cre*^ mice (described below) to generate mice heterozygous for both genes.

### METHOD DETAILS

#### Generation of *Ucn*^IRES–Cre:GFP^ mice

A cassette encoding IRES-mnCre:GFP was inserted just 3′ of the termination codon in the last coding exon of the *Ucn* gene. The 5′ arm (∼8 kb with *Spe*I and *Sal*I sites at 5′ and 3′ ends, respectively) and 3′ arm (∼1.5 kb with *Pme*I and *Not*I sites at 5′ and 3′ ends, respectively) of the *Ucn* gene were amplified from a C57BL/6 BAC clone by PCR using Q5 Polymerase (New England Biolabs) and cloned into polylinkers of a targeting construct that contained IRES-mnCre:GFP, a frt-flanked Sv40*Neo* gene for positive selection, and HSV thymidine kinase and *Pgk*-diphtheria toxin A chain genes for negative selection. The IRES-mnCre:GFP cassette has an internal ribosome entry sequence (IRES), a myc-tag and nuclear localization signals at the N terminus of Cre recombinase, which is fused to green fluorescent protein followed by an SV40 polyadenylation sequence (abbreviated here as IRES-Cre:GFP). The construct was electroporated into G4 ES cells (C57BL/6 × 129 Sv hybrid) and correct targeting was determined by Southern blot of DNA digested with *Xba*I using a ^32^P-labeled probe downstream of the 3′ arm of the targeting construct. Two of the 19 clones analyzed were correctly targeted. One clone that was injected into blastocysts resulted in good chimeras that transmitted the targeted allele through the germline. Progeny were bred with *Gt(Rosa)26Sor-FLP* recombinase mice to remove the frt-flanked SVNeo gene. Mice were then continuously backcrossed to C57BL/6 mice. Routine genotyping is performed with 3 primers: 5′ CAG CGA CGG GAC GAC C (*Ucn* forward), 5′ GCA TGC TTG TCT CTC CTA CCG (*Ucn* reverse) and 5′ GCT TCG GCC AGT AAC GTT AGG (IRES reverse). The wild-type allele gives a band of ∼400 bp, while the targeted allele gives a band of ∼255 bp after 34 cycles with 20-s annealing at 60C.

#### RNAscope fluorescence in situ hybridization (FISH)

Mice were deeply anesthetized with sodium pentobarbital and phenytoin sodium (0.3 mL, i.p.), decapitated, and brains rapidly dissected and frozen on crushed dry ice. Coronal sections (20 μm) were cut on a cryostat, mounted onto SuperFrost Plus slides, and stored at −80°C. RNAscope was performed following the manufacturer’s protocols. Images centered on the EW were acquired using a Keyence BZ-X710 microscope. FISH images were imported into Halo (Indica Labs) for automated probe quantification and cell counting.

#### Stereotaxic surgery

Mice were anesthetized with isoflurane (5% for induction, ∼2% for maintenance) and placed on a robotic stereotaxic frame (Neurostar). Eyes were protected with ophthalmic ointment. Rectal body temperature was maintained at 35–37°C using a feedback-controlled heating pad while on the stereotactic frame.

AAV1-Ef1a-DIO-synaptophysin:mCherry and AAV1-SYN1-DIO-YFP were mixed 1:1 and 0.5 μL was injected into the Edinger-Westphal nucleus (AP −3.55 mm, ML 0.0 mm, DV 3.8 mm) at a rate of 0.1 μL/min. For optogenetic experiments a prefabricated optical cannula comprising a bare optical fiber (Ø200 μm) and a ceramic ferrule (RWD) was implanted dorsal to the EW (AP −3.45mm, ML 0.0 mm, DV −3.35 mm). For fiber photometry experiments, a prefabricated optical cannula comprising a bare optical fiber (Ø400 μm) and a metal ferrule (Doric Lenses) was implanted just dorsolateral to the EW at a ±5° angle (AP −3.3 mm, ML ±0.15 mm, DV 3.8 mm). Both flat and angled+mirrored (same coordinates without the angle) optical fibers were used with no noticeable difference in mortality or functionality.

#### Immunohistochemical staining

At least 3 weeks after virus injection, mice were deeply anesthetized with sodium pentobarbital and phenytoin sodium (0.3 mL, i.p.) and intracardially perfused with ice-cold PBS followed by 4% PFA. Brains were post-fixed overnight in 4% PFA at 4°C, cryoprotected in 30% sucrose, frozen in OCT compound, and stored at −80°C. Coronal sections (40 μm) spanning the brain were cut on a cryostat and collected in cryoprotectant for long-term storage at −20°C. Although the AAV1 serotype has been shown to cross synapses in an anterograde direction, we never observed fluorescence in post-synaptic cells at the titer we used.

Sections were washed three times in PBS and incubated in a blocking solution (3% normal donkey serum and 0.2% Triton X-100 in PBS) for 1 h at room temperature. Sections were incubated overnight at 4°C in blocking solution with primary antibodies including: chicken-*anti*-GFP (1:10,000) and rabbit-*anti*-dsRed (1:2000). After three washes in PBS, sections were incubated for 1 h in PBS with secondary antibodies: Alexa Fluor 488 donkey anti-chicken and Alexa Fluor 594 donkey anti-rabbit, (1:500). Tissue was washed three times in PBS, mounted onto glass slides, and coverslipped with Fluoromount-G with DAPI (Southern Biotech).

Whole-slide fluorescent images were acquired using a Keyence BZ-X710 microscope. Images were minimally processed using Fiji to enhance brightness and contrast for optimal representation of the data. For the TIFF stacks, images were aligned using the BrainMaker workflow in NeuroInfo (MBF Bioscience).

#### Fiber photometry recording and analysis

Before every session, the implanted optic fiber was attached to a patch cord (Doric Lenses) using a ceramic sleeve (Doric Lenses). Recordings were acquired using an RZ5 BioAmp Processor from Tucker Davis Technologies (TDT). A 470-nm light-emitting diode (LED) (531 Hz, sinusoidal, Doric Lenses) was used to excite GCaMP6s, and a 405-nm LED was used as an isosbestic control (211 Hz, sinusoidal, Doric Lenses) using an LED driver (Doric Lenses) controlled via the RZ5. LED intensities were measured at the tip of the optic fiber to reach levels of 30–45 μW before being bandpass filtered (525 ± 25 nm; Doric, FMC4), transduced by a femtowatt silicon photoreceiver (Doric Lenses), and recorded by a real-time processor (TDT, RZ5). The 531- and 211-Hz signals were extracted in real time by the TDT program Synapse at a sampling rate of 1017 Hz. TTL pulses from behavioral tracking software (Ethovision) were used to synchronize the recordings for further analysis offline.

Given that the behavioral testing was performed in freely behaving animals there were relevant behavioral epochs whose 10 s pre/post bins overlapped. We removed epochs that occurred within 10 s of a previous epoch (that was included) from the dataset to prevent double counting overlapping fiber photometry signals. Custom Python scripts were used to extract and analyze fiber photometry signals at defined behavioral epochs. These scripts are available in the following Github repository: https://github.com/SabrinaYuY/Fiber_photometry_data_analysis and archived at Zenodo: https://doi.org/10.5281/zenodo.11624342. Where on/off artifacts were present, several seconds were excluded from the beginning and/or end of the trace. A 405-nm channel was used as a Ca^2+^-independent signal that captures autofluorescence, photobleaching, and motion artifacts. The 405-nm excitation signal was least-square linearly fitted to the 470nm signal to produce a scaled 405-nm signal. The fitted 405-nm signal was subtracted from the 470-nm signal to eliminate artifacts. Z scores were calculated based on the whole artifact-corrected 470-nm trace. *Z* score = (470-nm signal –mean 470-nm signal)/standard deviation of the entire 470-nm trace. To compute the area under the curve (AUC), the Trapezoidal Rule was used to approximate the area enclosed by the signal curve of the *Z* score trace and the time axis in time bins of 5 s.

#### Behavioral testing

##### Optogenetic activation

Lasers (Laserglow R471005GX) were used to deliver blue light pulses (473 nm, 10 ms pulse width, 5–10 mW) through a patch cable at a frequency of 20 Hz (A.M.P.I Master-8 or Agilent 33220A Arbitrary Waveform Generator). For most experiments the light stimulus was on for 2 s then off for 2 s to prevent desensitization. The 20-Hz stimulation was constant when mice were in the designated locations in proximity-triggered stimulus and real-time place aversion studies, for 2 min at a time in tail-suspension testing, and for 1min prior to each shock in foot-shock sensitivity testing. Control groups were a mix of Ai32^+/−^:*Ucn*^+/+^ mice that received light delivery and Ai32^+/−^:*Ucn*^+/*Cre*^ mice without light delivery.

##### Fast refeed

For both fiber photometry and optogenetic experiments mice were singly housed for at least 1 week and habituated to patch cable attachment 3+ times. Mice were placed into clean cages without access to chow at least 1hr before the start of the dark cycle. Approximately 15 h later, mice were connected to patch cables with light stimulation and, after 1–2 min, given access to a single pre-weighed chow pellet. Latency to first bite and pellet weight at 15 min and 30 min were recorded. Fiber photometry experiments were recorded for offline analysis (Ethovision).

##### Dehydrate rehydrate

For both fiber photometry and optogenetic experiments mice were singly housed in cages with water provided through external sipper ports for at least 5 days and habituated to patch cable attachment 3+ times. Water sippers were removed at least 1hr before the start of the dark cycle. Approximately 15 h later mice were connected to patch cables with light stimulation and, after 1–2 min, a single pre-weighed water sipper was inserted through an external sipper port. Latency to first lick and bottle weight at 5, 15, and 30 min were recorded. Fiber photometry experiments were recorded for offline analysis (Ethovision).

##### Novelty-induced hypophagia

For both fiber photometry and optogenetic experiments mice were singly housed in cages with external sipper ports for at least 5 days and habituated to patch cable attachment 3+ times. Access to Ensure was provided through an external sipper port for 30 min once per day for at least 4 days. On the test day mice were attached to patch cables with light stimulation and placed under bright light in a novel, completely empty sipper cage. After about 1 min a single pre-weighed sipper bottle containing Ensure was inserted through an external sipper port. Latency to first lick and bottle weight at 5, 15, and 30 min were recorded. Fiber photometry experiments were recorded for offline analysis (Ethovision). In additional permutations of this test, optogenetic stimulation was performed during the 1^st^ Ensure exposure in the home cage (in order to isolate the food neophobia component) or in the home cage at least one week after the completion of the above timeline (to isolate the hedonic consumption component, while excluding any anxiogenic components).

##### Tail-suspension test

Mice were attached to patch cables for light delivery and tails were threaded through smooth plastic cylinders to prevent tail-climbing. Mice were then quickly suspended by the tail (via lab tape placed approximately 1cm from the distal end of the tail) > 40 cm from the ground. In optogenetic experiments, after a 2 min baseline period, laser stimulation alternated between on and off every 2 min for the duration of the 6-min test. Experiments were recorded for offline analysis (Ethovision). For optogenetic experiments, Ethovision mobility tracking was used to measure periods of immobility, mobility, and high mobility. Mobility tracking calculates periods when the complete area detected as the animal is changing, even if the center point does not move. The thresholds between mobility types were set manually to match experimenter observations as closely as possible. For fiber photometry experiments mobility was handscored using Ethovision’s manual scoring interface. Periods of whole-body movements were classified as intense struggling bouts while any smaller movements involving only upper or lower body movements were classified simply as movement.

##### Foot-shock sensitivity

Mice were attached to patch cables and placed into a 28 × 28 × 25 cm chamber with a floor of multiple metal rods connected to a shock grid (Med Associates). House lights turned on at the start of the experimental protocol, 120 s later the conditioned stimulus (CS; light and tone) was presented for 17 s every 90 s. The 2-s unconditioned stimulus (US; footshock of variable intensity) occurred during the last 2 s of the CS. For optogenetic experiments, 9 CS-US pairings were presented: 0.07, 0.07, 0.15, 0.2, 0.3, 0.4, 0.5, 0.15, 0.3 mA. No optogenetic activation occurred prior to the first pairing, for the other 8 pairings, a laser was used to deliver 5–10 mW blue light (473 nm) at 20 Hz (10 ms pulse width) for 65 s beginning 60 s prior to each US. For fiber photometry experiments, 8–10 CS-US pairings were presented (terminal intensity was determined by behavioral response, so if mice jumped and vocalized at 0.3 mA, they did not progress to 0.4 mA). An additional 4 US were presented without CS. US presentation with CS was: 0.07, 0.15, 0.2, 0.3, 0.4, 0, 0.4, 0.3, 0.3, 0.15, 0.07 mA. Uncued US were then presented at least 90 s apart: 0.15, 0.3, 0.3, 0.15 mA. Behavior was recorded for offline analysis (Ethovision).

##### Repeated restraint

Mice were singly housed. After at least 1 week of habituation to being singly housed, mice and chow were weighed daily in the first 4 h of the light cycle through the rest of the protocol. On behavior testing and stress days, weights were always recorded prior to any manipulation. After at least 1 week, in the first 4 h of the light cycle each day for 14 days, mice were connected to patch cables for light delivery, scruffed, and placed in a flexible, clear plastic cone that was taped and tied closed. After 30 min, mice were returned to their home cages. The restraint stress was recorded for offline analysis of struggling behavior. For optogenetic experiments Ethovision mobility tracking was used to measure periods of immobility, mobility, and high mobility. Mobility tracking calculates periods when the complete area detected as the animal is changing, even if the center point does not move. The thresholds between mobility types were set manually to match experimenter observations as closely as possible. For fiber photometry experiments mobility was hand-scored using Ethovision’s manual scoring interface. Periods of whole body movements were classified as intense struggling bouts while smaller movements involving only upper or lower body movements were classified as small mobility bouts. Approximately 2 h after the first restraint, mice were tested in the elevated plus maze. Approximately 4 h after the second restraint, mice were tested in the open field test. Approximately 22 h after the 8th and 9th restraints, mice were tested in the forced swim test. Body and chow weights were recorded intermittently in the first 4 h of the light cycle during a 3 wk recovery period after the 14^th^ day of the restraint paradigm. Experiments were recorded for offline analysis (Ethovision).

##### Elevated plus maze

Mice were placed individually in the center of an elevated maze (65 cm above the floor) with 2 open and 2 closed arms (∼30 cm) joined together by a square center zone. The closed arms were enclosed by tall, opaque walls on 3 sides. Experiments were recorded for 10 min for offline analysis (Ethovision). 10 min were recorded in case being attached to the patch cable inhibited exploration and to allow for the possibility that optogenetic stimulation of this putatively exclusively peptidergic population would be most effective after several minutes of stimulation.

##### Open-field test

Mice were placed individually in the periphery of a novel open arena measuring 40 cm square and recorded for 30 min for offline analysis using Ethovision. 30 min were recorded in order to test for effects on anxiety-like behavior and locomotor behavior.

##### Scruff position

Mice were connected to patch cables and allowed to explore an arena for several minutes, then briefly picked up by the tail, placed on a cage top and scruffed. Scruffed mice were held approximately 45 cm above the floor oriented belly up for ∼10 s, rotated sideways for ∼10 s, rotated belly down for ∼10 s, then returned to the arena floor. Experiments were recorded for offline analysis (Ethovision).

##### Conditioned taste avoidance (CTA)

Mice were individually housed in cages with external angled ports for insertion of 2 test tubes with sippers. Mice were habituated to patch cable attachment in the home cage for a total of at least 60 min for 3 days. After at least 5 days of acclimation to the cages and sippers, mice were water deprived overnight. On days 1–3, mice had *ad libitum* access to one water tube for 30 min of the first 4 h and last 2 h of the light cycle. The side used for water delivery was alternated. On day 4, mice were attached to the patch cable and given 30 min access to a novel 5% sucrose solution after which they received photostimulation (20 Hz, 5–10 mA, 2 s on, 2 s off) for 30 min. Water access was provided for 1 h in the afternoon. On day 5 mice had water access following the same schedule as days 1–3. Days 6 and 7 followed the same conditioning schedule as day 4. On day 8, mice were given access to water and 5% sucrose for 30 min and consumption of each was calculated by subtracting the session end tube weight from the session start tube weight. Sucrose preference during the test on day 8 was calculated as: grams of sucrose consumed/(grams of sucrose + grams of water consumed) × 100.

##### Food neophobia

Mice were attached to patch cables and returned to their home cages. One pre-weighed novel high-fat, high-sugar food pellet (Research Diets D12266B) was added to the floor of the home cage. Latency to first bite and pellet weight at 5, 15, 30, and 60 min was recorded.

##### Stimulation near sipper

Mice were attached to patch cables and placed in a brightly lit empty cage with a sipper port at one end. Laser stimulation was triggered by entry into a pre-defined sipper zone spanning the full width of the cage, beginning ∼8 cm from the sipper port (Ethovision).

##### Real-time place avoidance

Mice were attached to patch cables and placed into a two-chamber arena to freely explore for 40 min. The two chambers were distinguishable by both floor texture and visual cues. After a 10-min baseline period, laser stimulation was triggered while mice resided in one side of the chamber, but not the other side (laser-paired side was counterbalanced). After 20 min, the laser was no longer active. Ethovision was used for real-time animal tracking to trigger laser stimulation, and for further offline analysis.

### QUANTIFICATION AND STATISTICAL ANALYSIS

Data are presented as the mean ± standard error of the mean (SEM), except violin plots on which the solid central line represents the median, the two dotted lines represent quartiles, and connecting lines across plots track specific individuals. Ns reported in figures are animal numbers. Data were analyzed for statistical significance using GraphPad Prism. For data with only 2 groups a two-tailed t test was used. For data with more than 2 groups, standard or repeated measures one- or two-way ANOVA was used, as appropriate. The asterisks in the figures represent the *p* values as follows: *p* < 0.05*, *p* < 0.01**, *p* < 0.001***, *p* < 0.0001****. More details regarding the specific statistical test used and additional details about sample size, sex, control types, and number of epochs in each fiber photometry experiment are included in Table S1. No statistical methods were used to predetermine sample sizes, but our sample sizes are similar to those reported in previous publications. Outliers were identified by ROUT test with Q = 1%.

Data were collected and analyzed by an investigator blinded to experimental condition. Animals with mistargeted implants outside the EW were excluded from analyses. Littermates were randomly assigned to experimental groups, and animals were tested in a random order across all biological replicates. Data for *in situ* hybridization was pooled from at least two animals.

## Supplementary Material

1

SUPPLEMENTAL INFORMATION

Supplemental information can be found online at https://doi.org/10.1016/j.celrep.2025.115814.

## Figures and Tables

**KEY RESOURCES TABLE:** 

REAGENT or RESOURCE	SOURCE	IDENTIFIER

Antibodies		
Rabbit-*anti*-dsRed	Takara	Cat # 632496; RRID: AB_10013483
Chicken-*anti*-GFP	Abcam	Cat# 13970; RRID: AB_300798
Alexa Fluor 594 donkey-*anti*-rabbit	Jackson ImmunoResearch	Cat #715-585-150; RRID: AB_2340854
Alexa Fluor 488 donkey-*anti*-chicken	Jackson ImmunoResearch	Cat #703-545-155; RRID: AB_2340375

Bacterial and virus strains		

pAAV1-hSyn-DIO-YFP	Palmiter Lab	N/A
pAAV1-Ef1a-DIO-Syn:mCherry	Palmiter Lab	N/A

Chemicals, peptides, and recombinant proteins		

Paraformaldehyde 32% Aqueous Solution	Electron Microscopy Sciences	Cat #15714-S
Normal donkey serum	Jackson ImmunoResearch	Cat #017-000-121, RRID: AB_2337258
O.C.T.	Thermofisher	Cat #23-730-571
DAPI Fluoromount-G	SouthernBiotechne	Cat #0100-20
C&B Metabond	Parkell	SKU: S380
Euthasol	Virbac	Ref# 200-071
Dental Cement	A-M Systems	Ref# 594845

Critical commercial assays		

RNAscope Fluorescent Multiplex Assay V1	ACD Biotechne	Discontinued

Deposited data		

Immunohistochemical characterization of a gene-targeted UcnCre line of mice	This study	Zenodo data: https://doi.org/10.5281/zenodo.11492903

Experimental models: Organisms/strains		

Mouse: *Rosa26*^*LSL*^*-*^*tdTomato*^	Jackson Laboratory	Strain# 7914
Mouse: *Rosa26*^*LSL*^*-*^*ChR2*^	Jackson Laboratory	Strain# 24109
Mouse: TIGRE^*LSL*^*-*^*GCaMP6s*^	Jackson Laboratory	Strain# 31562
Mouse: *Ucn*^*IRES*^*-*^*Cre:GFP*^	This study	N/A

Oligonucleotides		

5′ CAG CGA CGG GAC GAC C (Ucn forward)		N/A
5′ GCA TGC TTG TCT CTC CTA CCG (Ucn reverse)		N/A
5′ GCT TCG GCC AGT AAC GTT AGG (IRES reverse)		N/A

Software and algorithms		

Prism	GraphPad Software	www.graphpad.com
Fiji/ImageJ	Fiji/NIH	www.fiji.sc
Original Code for the analysis of fiber photometry data	This study	Zenodo: https://doi.org/10.5281/zenodo.11624342 and Github: https://github.com/SabrinaYuY/Fiber_photometry_data_analysis/
Ethovision	Noldus Technology	www.noldus.com
Synapse	Tucker-Davis Technologies	https://www.tdt.com/component/synapse-software/
NeuroInfo	MBF Bioscience	https://www.mbfbioscience.com/products/neuroinfo
Halo	Indica Labs	https://indicalab.com/halo/

Other		

Cryostat	Leica	Ref #CM1950
Fluorescence Microscope	Keyence	Ref# BZ-X710
473 nm Laser	LaserGlow	R471005GX
Master-8 Pulse Stimulator	A.M.P.I.	N/A
Arbitrary Waveform Generator	Agilent	33220A
Low-autofluorescence Mono Fiber-optic Patch Cords – 200um fiber core diameter	Doric Lenses	Ref# MFP_200
Fiber Optic Cannulae	RWD	Cat# R-FOC-BL200C-22NA
Foot-shock chambers	Med Associates	
RZ5P BioAmp Processor	Tucker Davis Technologies	N/A
RNAscope Probe Mm-*Ucn*	ACD Biotechne	Cat#: 466261
RNAscope Probe Mm-tdTomato	ACD Biotechne	Cat#: 317041
RNAscope Probe Mm-*Cck*	ACD Biotechne	Cat#: 402271
RNAscope Probe Mm-*Adcyap1*	ACD Biotechne	Cat#: 405911
RNAscope Probe Mm-*Slc17a6*	ACD Biotechne	Cat#: 319171
RNAscope Probe Mm-*Tac1*	ACD Biotechne	Cat#: 410351
Immunohistochemical characterization of a gene-targeted Ucn^Cre^ line of mice	This study	Zenodo: https://doi.org/10.5281/zenodo.11492903

## Highlights

- Hedonic consumption elicits a biphasic pattern of Ucn^EW^ activity
- Ucn^EW^ optogenetic activation inhibits hedonic and homeostatic consumption
- Ucn^EW^ activity increases during active coping but does not scale with intensity
- Coping behavior and stress outcomes are minimally impacted by Ucn^EW^ activation
