# Supplementary material for: Edinger-Westphal Urocortin-1 neurons regulate consumption and affect
Source: Cell Rep. Author manuscript; Available in PMC 2025 Oct 20. (PMC12536611; doi:10.1016/j.celrep.2025.115814)
Supplement: 1 [file NIHMS2113350-supplement-1.pdf]

Cell Reports, Volume *44*

Supplemental information

Edinger-Westphal Urocortin-1 neurons regulate consumption and affect

Rebecca J. Bluett, Ying Yu, Jordan L. Pauli, Carlos A. Campos, and Richard D. Palmiter

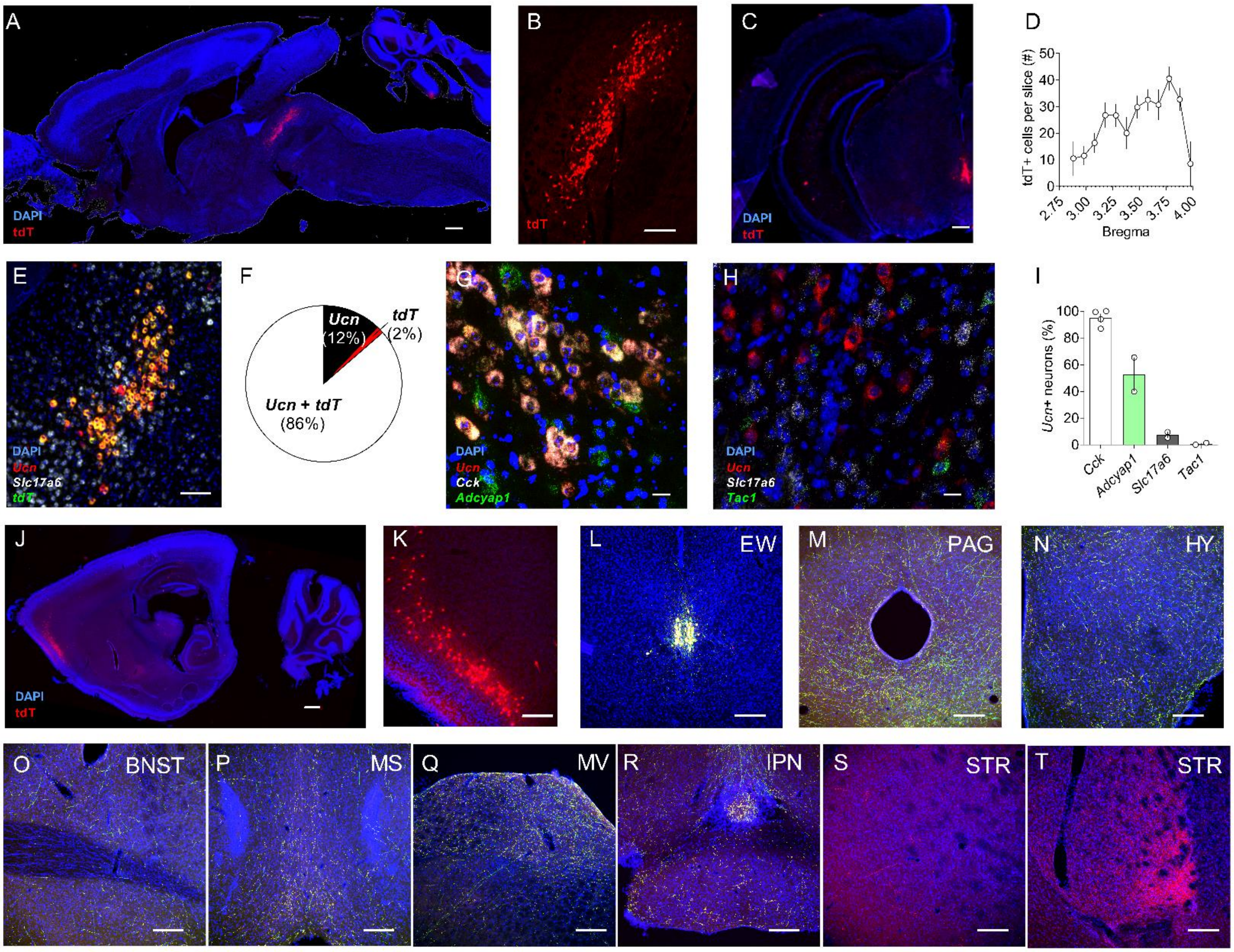

Figure S1. Characterization of a gene-targeted *Ucn*^{Cre} line of mice

(A) Representative image of immunohistochemistry for tdTomato (red) in the Edinger Westphal of a Ucn$^{Cre/+}$::tdT$^{+/-}$ mouse, sagittal slice. Scale bar 500 µm.
(B) Representative image of immunohistochemistry of tdTomato (red) in the Edinger Westphal of a Ucn$^{Cre/+}$::tdT$^{+/-}$mouse, sagittal slice. Scale bar 200 µm.
(C) Representative image of immunohistochemistry of tdTomato (red) in the Edinger Westphal of a Ucn$^{Cre/+}$::tdT$^{+/-}$mouse, coronal slice.
(D) Average count of tdT-expressing cells across Bregma levels of the Edinger Westphal nucleus.
(E) Representative image of FISH staining for *Ucn* (red), *tdT* (green) and *Slc17a6* (Vglut2; white). Scale bar 200 µm.
(F) Quantification of FISH staining.
(G) Representative image of FISH staining for *Ucn* (red), *Adcyap1* (PACAP; green) and *Cck* (white). Scale bar 20 µm.
(H) Representative image of FISH staining for *Ucn* (red), *Tac1* (yellow) and *Slc17a6* (white). Scale bar 20 µm.
(I) Quantification of FISH co-localization of *Ucn* with *Cck*, *Adcyap1*, *Slc17a6*, and *Tac1*.
(J) Representative image of immunohistochemistry of tdTomato (red) in the insular cortex of a Ucn$^{Cre/+}$::tdT$^{+/-}$mouse, sagittal slice. Scale bar 500 µm.
(K) Higher magnification of tdT+ cells in (J). Scale bar 200 µm.
(L) Representative image of immunostaining for YFP and tdT in Edinger-Westphal of Ucn$^{Cre/+}$ mouse injected with AAV1-hSyn-DIO-YFP and AAV1-Ef1a-DIO-synaptophysin:mCherry. Scale bar 200 µm.
(M) Representative image of immunostaining of projections in the periaqueductal gray (PAG) from neurons in (L). Scale bar 200 µm.
(N) Representative image of immunostaining of projections in the hypothalamus (HY) from neurons in (L). Scale bar 200 µm.
(O) Representative image of immunostaining of projections in the bed nucleus of the stria terminalis (BNST) from neurons in (L). Scale bar 200 µm.
(P) Representative image of immunostaining of projections in the medial septum (MS) from neurons in (L). Scale bar 200 µm.
(Q) Representative image of immunostaining of projections in the medial vestibular nucleus (MV) from neurons in (L). Scale bar 200 µm.
(R) Representative image of immunostaining of projections in the interpeduncular nucleus (IPN) from neurons in (L). Scale bar 200 µm.
(S) Representative image of immunostaining of projections in the dorsal striatum (STR) from neurons in (L). Scale bar 200 µm.
(T) Representative image of immunostaining of projections in the dorsal striatum (STR) from a Ucn$^{Cre/+}$::tdT$^{+/-}$ mouse. Scale bar 200 µm.

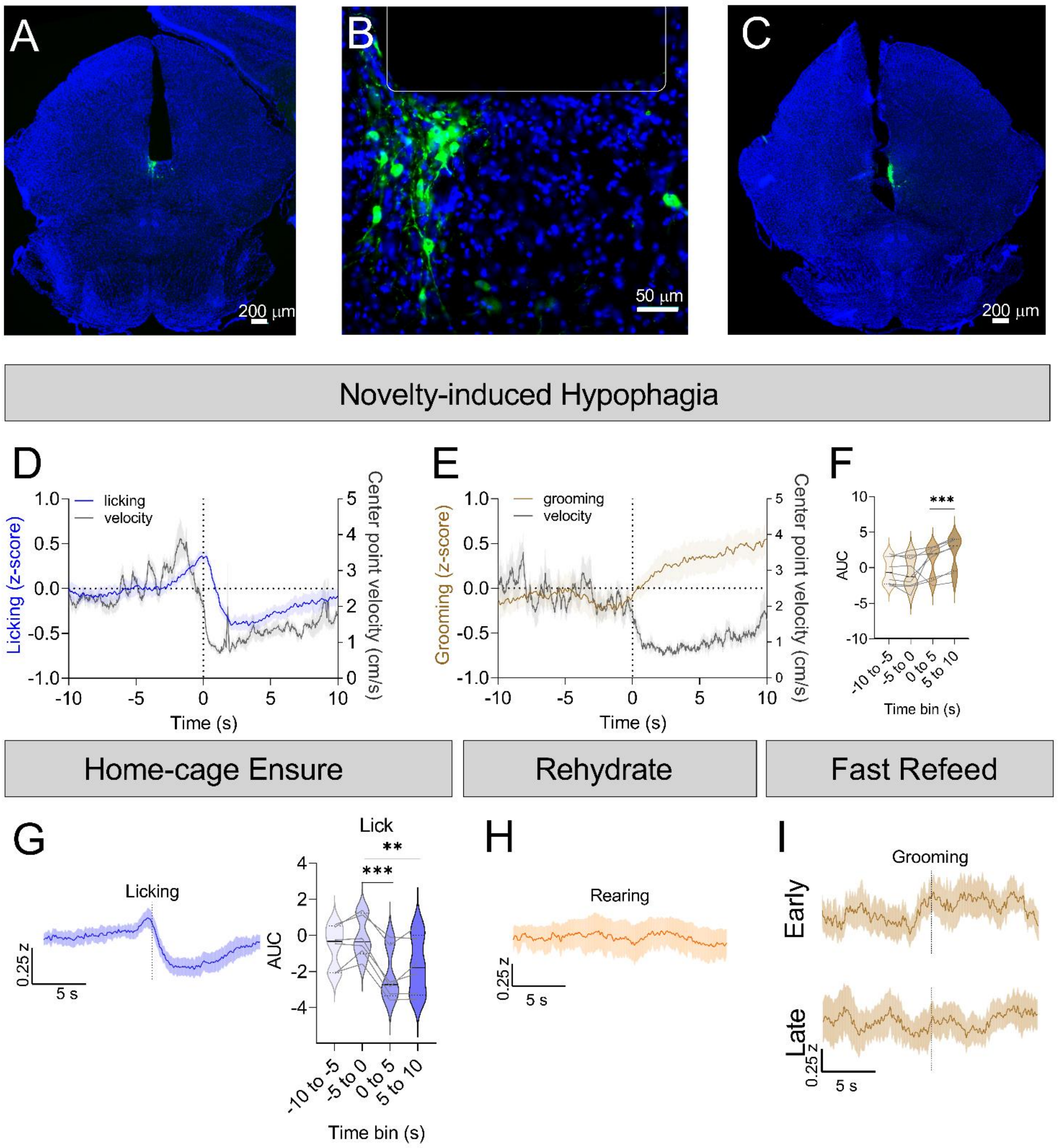

Figure S2. Ucn^{EW} calcium activity is not directly related to motor behavior, related to Figure 1.
(A) Representative image of immunohistochemistry for GFP (green) in a $Ucn^{Cre/+}$::$Ai162^{+/-}$ mouse with a flat fiber optic cannula placed just above the Edinger Westphal. Scale bar 200 µm.
(B) Higher magnification of immunohistochemistry in (A). Scale bar 50 µm.
(C) Representative image of immunohistochemistry for GFP (green) in a $Ucn^{Cre/+}$::$Ai32^{+/-}$ mouse with an angled, mirror fiber optic cannula placed adjacent to the Edinger-Westphal. Scale bar 200 µm.
(D) Peri-event plots of 10 s before and after lick initiation during the NIH test with fiber photometry z-score in blue and mouse velocity, tracked by Ethovision, in grey.
(E) Peri-event plots of 10 s before and after grooming initiation during the NIH test with fiber photometry z-score in brown and mouse velocity, tracked by Ethovision, in grey.
(F) Area under the curve of z-scores in (E).
(G) (Left) Fiber photometry z-score, peri-event plot of 10 s before and after lick initiation in the home-cage during the 2nd Ensure exposure. (Right) Area under the curve of z-scores in Left.
(H) Fiber photometry z-score, peri-event plot of 10 s before and after rearing initiation during rehydration period after overnight water deprivation.
(I) Fiber photometry z-score, peri-event plots of 10 s before and after grooming initiation from 0-15 min (top, 'Early') and 15-30 min (bottom, 'Late') during refeeding after an overnight fast.

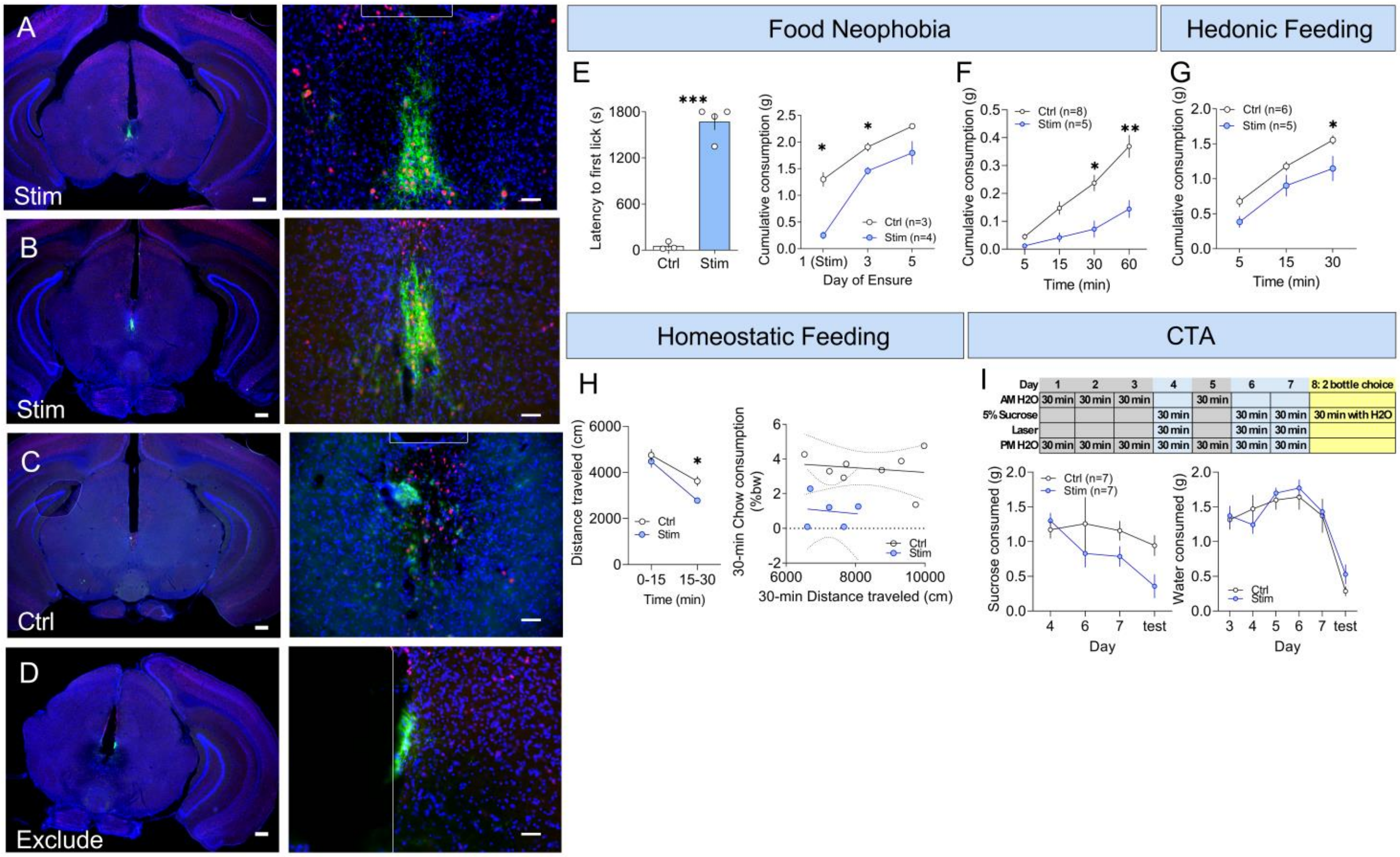

Figure S3. Optogenetic activation of Ucn^{EW} neurons reduces consumption, related to Figure 2.

(A & B) (Left) Representative images of immunohistochemistry for GFP (green) and Fos (red) in a $\text{Ucn}^{Cre/+}$::$\text{Ai32}^{+/-}$ mouse with a fiber optic cannula placed above the Edinger Westphal. (Right) Magnification of (left).

(C) (Left) Representative image of immunohistochemistry for GFP (green) and Fos (red) in a $\text{Ucn}^{+/+}$::$\text{Ai32}^{+/-}$ mouse with a fiber optic cannula placed above the Edinger Westphal. (Right) Magnification of (left).

(D) (Left) Representative image of immunohistochemistry for GFP (green) and Fos (red) in an excluded $\text{Ucn}^{Cre/+}$::$\text{Ai32}^{+/-}$ mouse with an improperly placed fiber optic cannula. (Right) Magnification of (left).

(E) (Left) Latency to first lick during the first Ensure exposure with optogenetic stimulation of Ucn^{EW} neurons. (Right) Total home cage Ensure consumption during the 1st, 3rd, and 5th exposure after optogenetic stimulation only during the 1st exposure.

(F) Home-cage cumulative consumption of novel high-fat, high-sugar food pellet.

(G) Cumulative consumption of familiar Ensure in the home cage with optogenetic stimulation of Ucn^{EW} neurons.

(H) (Left) Time course of distance traveled during the 30-min refeeding period after an overnight fast. (Right) Correlation of chow consumption and distance traveled in the 30-min fast refeed test.

(I) (Top) Timeline of Conditioned Taste Avoidance (CTA) paradigm. (Bottom left) Consumption of 5% sucrose solution during the CTA paradigm. (Right) Total daily water consumption during the CTA paradigm.

(A-D left) Scale bar 200 µm.

(A-D right) Scale bar 200 µm.

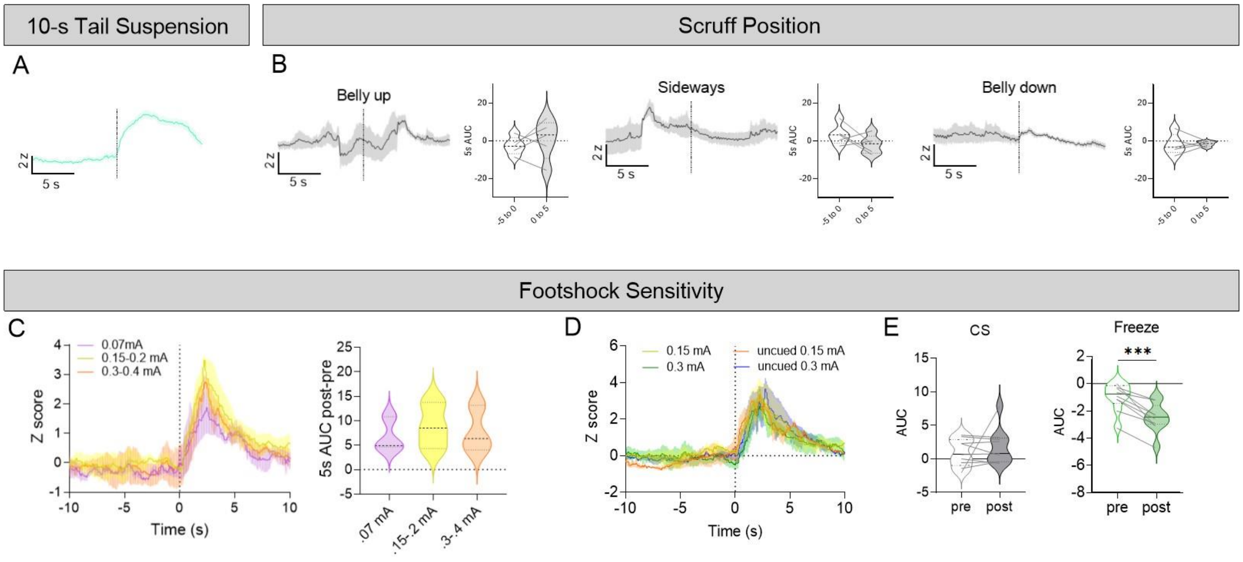

Figure S4. Ucn^{EW} calcium activity does not depend on body position or scale with shock intensity, related to Figure 3.
(A) Fiber photometry z-score peri-event plots of 10 s before and after initiation of a 10 s tail suspension.
(B) Fiber photometry z-score peri-event plots and AUC of z-score before and after (left) scruffed mouse moved into belly up position ~50 cm above arena floor (middle) mouse turned sideways, (right) mouse turned belly down.
(C) Fiber photometry (left) z-score peri-event plots of 10 s before and after initiation of a 2 s footshock of various intensities and (right) AUCs for the 5 s after footshock minus the 5 s before footshock.
(D) Fiber photometry z-score peri-event plots of 10 s before and after initiation of a 2-s footshock comparing cued and uncued shocks of the same intensity.
(E) Area under the curve of z-scores in Figure 3C (right).

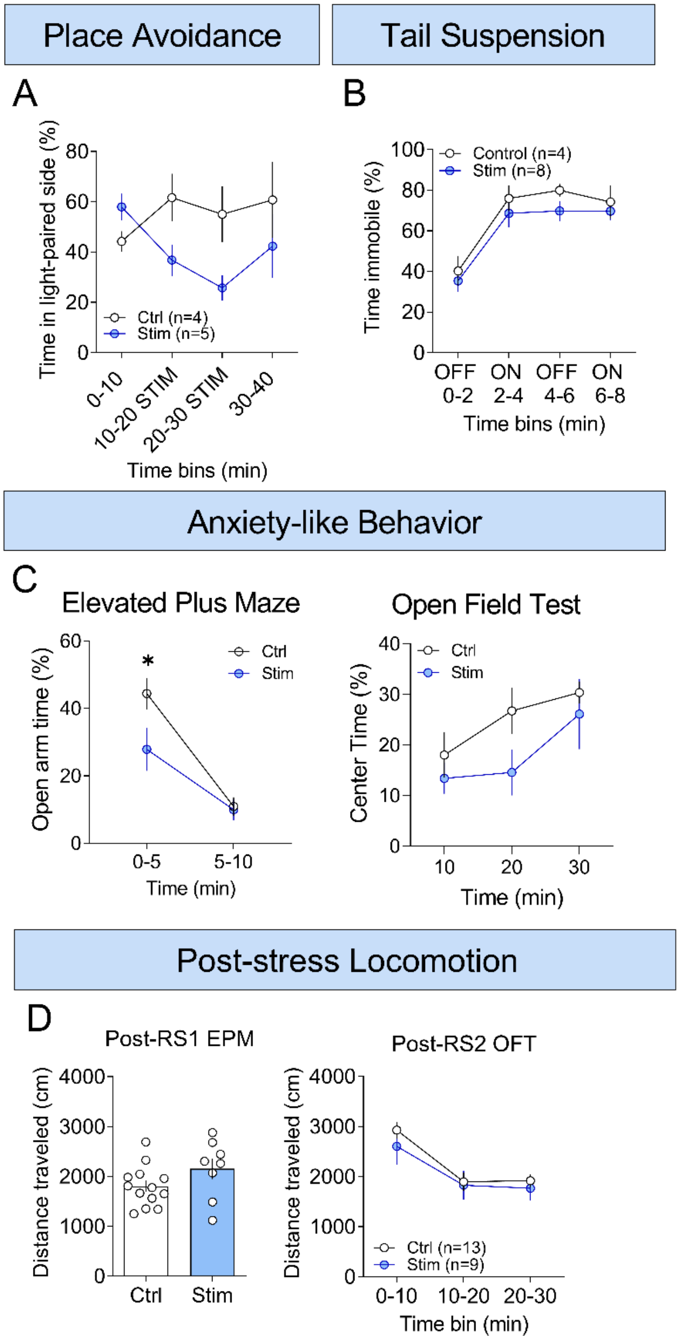

Figure S5. Optogenetic activation of Ucn^{EW} neurons is mildly aversive, related to Figure 4.
(A) Percent time, in 10-min bins, in light-paired side of a chamber with 2 sides with distinct floor textures and visual cues. No light stimulation occurred during the baseline 10 min. From 10-30 min, optogenetic stimulation was triggered anytime mice were in the light-paired side.
(B) Time highly mobile in the tail suspension test across 4 2-min bins, with laser stimulation on from minute 2-4 and 6-8.
(C) Time in the closed arms of the elevated plus maze in stress-naïve mice with optogenetic stimulation compared to controls.
(D) (left) Distance traveled during the post-RS1 elevated plus maze. (right) Distance traveled, by 10-min bin, in the post-RS2 open field test.

Supplementary Table 1: Statistics and experiment details

Figure	Experiment Details	Statistical Tests	Main Effect Results (P value)	Multiple Comparisons	Outliers
Fig. 1C	N = 9 (5 male, 4 female); data points represent the average of >10 lick epochs and >5 approach epochs per animal	Repeated Measures (RM) one-way ANOVA: **Lick** F (1.603, 12.83) = 23.80;**Approach** F (1.365, 10.92) = 9.024	**Lick**: P<0.0001 ; **Approach** P=0.0082	Tukey's MCT **Lick**: (-5 to 0) vs (0 to 5) P=0.0003, (-10 to -5) vs (0 to 5) P=0.036, (-10 to -5) vs (-5 to 0) P=0.007 ;**Approach**: (-5 to 0) vs (0 to 5) P=0.0399, (-10 to -5) vs (0 to 5) P=0.0282, (-10 to -5) vs (-5 to 0) P=0.1629	
Fig. 1F	N = 7 (5 male, 2 female); data points represent the average of >5 lick epochs and 2-23 approach epochs per animal	RM one-way ANOVA: **Lick** F (1.394, 8.363) = 4.374; **Approach** F (1.852, 11.11) = 0.5306	**Lick**: P=0.0598 ; **Approach** P=0.5892	Tukey's MCT **Lick**: (-5 to 0) vs (0 to 5) P=0.0193, (-10 to -5) vs (0 to 5) P=0.1461, (-10 to -5) vs (-5 to 0) P=0.9459 ;**Approach**: (-5 to 0) vs (0 to 5) P=0.7435, (-10 to -5) vs (0 to 5) P=0.9425, (-10 to -5) vs (-5 to 0) P=0.5981	Lick: ROUT Q=1% no outliers found
Fig. 1H	N = 7 (4 male, 3 female); data points represent the average of >15 epochs per animal	RM one-way ANOVA: **Early** F (1.241, 7.445) = 2.612; **Late** F (1.578, 9.466) = 7.496	**Early**: P=0.1460 ; **Late** P=0.0143	Tukey's MCT **Early**: (-5 to 0) vs (0 to 5) P=0.2014, (-10 to -5) vs (0 to 5) P=0.3809, (-10 to -5) vs (-5 to 0) P=0.8342 ;**Late**: (-5 to 0) vs (0 to 5) P=0.0088, (-10 to -5) vs (0 to 5) P=0.9907, (-10 to -5) vs (-5 to 0) P=0.0384	
Fig. 1J	N = 7 (4 male, 3 female); data points represent the average of 4-91 early epochs and 11-115 late epochs per animal	RM one-way ANOVA: **Early** F (1.619, 9.714) = 0.2801; **Late** F (1.437, 8.623) = 2.100	**Early**: P=0.7169 ; **Late** P=0.1836	Tukey's MCT **Early**: (-5 to 0) vs (0 to 5) P=0.9917, (-10 to -5) vs (0 to 5) P=0.8246, (-10 to -5) vs (-5 to 0) P=0.7276 ;**Late**: (-5 to 0) vs (0 to 5) P=0.4398, (-10 to -5) vs (0 to 5) P=0.2581, (-10 to -5) vs (-5 to 0) P=0.8304	
Fig. 2A	**Ctrl** (cre-, light+) N = 11 (7 male, 4 female); **Stim** N = 9 (4 male, 5 female)	Two-way RM ANOVA: **Time x Group** F (2, 36) = 3.718; **Time** F (2, 36) = 119.8;**Group** F (1, 18) = 8.881	**Time x Group**: P=0.0341;**Time**: P<0.0001;**Group**: P=0.0080	Bonferroni's MCT 0-5 min: P=0.9145 ; 0-15min: P=0.0203; 0-30 min: P=0.0021	
Fig. 2B	**Ctrl** (cre-, light+) N = 11 (7 male, 4 female); **Stim** N = 9 (4 male, 5 female)	Two-tailed Unpaired Welch's t test	P=0.0077		
Fig. 2C	**Ctrl** (9cre-, light+; 3 no light[†]) N = 12 (5 male, 7 female); **Stim** N = 12 (4 male, 8 female)	Two-way RM ANOVA: **Time x Group** F (2, 44) = 4.658; **Time** F (1.168, 25.70) = 51.98;**Group** F (1, 22) = 6.953	**Time x Group**: P=0.0146;**Time**: P<0.0001;**Group**: P=0.0151	Bonferroni's MCT 0-5 min: P=0.0916; 0-15min: P=0.0708; 0-30 min: P=0.03	See Fig. 2D below
Fig. 2D	**Ctrl** (9cre-, light+; 3 no light[†]) N = 12 (5 male, 7 female); **Stim** N = 12 (4 male, 8 female)	Two-tailed Unpaired Welch's t test	P=0.0052		ROUT Q=1% found 1 outlier in ctrl group which was excluded from all rehydrate data (2C & 2D)
Fig. 2E	**Ctrl** (10cre-, light+; 2 sham implant[††]) N = 12 (6 male, 6 female); **Stim** N = 13 (5 male, 8 female)	Two-way RM ANOVA: **Time x Group** F (1, 23) = 22.07; **Time** F (1, 23) = 102.5;**Group** F (1, 23) = 21.62	**Time x Group**:P<0.0001;**Time**: P<0.0001;**Group**: P=0.0001	Bonferroni's MCT 0-15min: P=0.0157; 0-30 min: P<0.0001	
Fig. 2F	**Ctrl** (10cre-, light+; 2 sham implant[††]) N = 12 (6 male, 6 female); **Stim** N = 13 (5 male, 8 female)	Unpaired Welch's t test	P=0.0171		
Fig. 2G	**Ctrl** (2cre-, light+; 2 sham implant[††]) N = 4 (2 male, 2 female); **Stim** N = 8 (4 male, 4 female)	Two-way RM ANOVA: **Time x Group** F (2, 20) = 2.524; **Time** F (2, 20) = 0.6067;**Group** F (1, 10) = 0.1017	**Time x Group**: P=0.1053;**Time**: P=0.5549;**Group**: P=0.7564	Bonferroni's MCT 0-15min: P>0.9999; 0-30 min: P>0.9999; 0-60 min: P=0.5128	
Fig. 2H	**Ctrl** (7cre-, light+) N =7 (3 male, 4 female); **Stim** N = 7 (2 male, 5 female)	Two-tailed Unpaired Welch's t test	P=0.03		
Fig. 3B	N = 11 (4 male, 7 female); data points represent the average of >5 stop epochs, >10 start epochs per animal, and 4-11 intense epochs per animal	Ordinary one-wa y ANOVA: F (2, 30) = 23.80	P<0.0001	Tukey's MCT: Stop vs Start: P <0.0001; Stop vs Intense: P <0.0001; Start vs Intense P=0.2111	
Fig. 3D	N =9 (5 male, 4 female); data points represent the average of >5 cs epochs, >10 freeze epochs, 0-6 skitter epochs; 1-9 run epochs; and 1-6 jump epochs per animal	Brown-Forsythe ANOVA test: F (4, 23.23) = 20.73	P<0.0001	Dunnett's MCT: CS vs Freeze P=0.0318; CS vs Jump P=0.001; CS vs Skitter P=0.01; CS vs Run P=0.0036; Freeze vs Run P=0.001; Freeze vs Jump P=0.0003; Skitter vs Run P>0.9999; Skitter vs Jump P=0.9943; Run vs Jump P=0.9993	ROUT Q=1%: none found
Fig. 3F	N = 5 (3 male, 2 female); data points represent the average of >10 epochs	Two-tailed paired t tests	RS1 intense P= 0.006; RS3 intense P= 0.0384; RS14 intense P=0.054		

Fig. 3H	N = 5 (3 male, 2 female); data points represent the average of >5 rs1 epochs, 3-21 rs3 epochs, and 2-13 rs14 epochs	Two-tailed paired t tests	RS1 mobile P= 0.0094; RS3 mobile P= 0.0635; RS14 mobile P= 0.414		
Fig. 4A	**Ctrl** (cre-, light+) N =4 (3 male, 1 female); **Stim** N = 5 (2 male, 3 female)	Two-tailed unpaired t test	P=0.0249		
Fig. 4B	**Ctrl** (cre-, light+) N =4 (1 male, 3 female); **Stim** N = 8 (4 male, 4 female)	Two-tailed unpaired t test	P=0.0472		
Fig. 4C	**Ctrl** (cre-, light+) N =4 (3 male, 1 female); **Stim** N = 5 (2 male, 3 female)	Two-way RM ANOVA: **Time x Group** F (5, 35) = 0.7160; **Time** F (5, 35) = 38.49; **Group** F (1, 7) = 2.614	**Time x Group**: P=0.6156; **Time**: P<0.0001; **Group**: P=0.1499	Bonferroni's MCT: .07 P>0.9999; .15 P>0.9999; .2 P=0.3606 ;.3 P>0.9999; .4 P=0.6891; .5 P=0.6891	
Fig. 4D	**Ctrl** (cre-, light+) N =4 (3 male, 1 female); **Stim** N = 5 (2 male, 3 female)	Two-tailed unpaired t test	P=0.1996		
Fig. 4F	**Ctrl** (9cre-, light+; 4 no light[†]) N =13 (6 male, 7 female); **Stim** N = 9 (3 male, 6 female)	RM mixed-effects analysis: Time x Group F (13, 258) = 0.6081; Time F (5.314, 105.5) = 1.050; Group F (1, 20) = 0.08357	**Time x Group**: P=0.8467; **Time**: P=0.3942; **Group**: P=0.7755		
Fig. 4G	**Ctrl** (9cre-, light+; 4 no light[†]) N =13 (6 male, 7 female); **Stim** N = 9 (3 male, 6 female)	Two-way RM ANOVA: **Time x Group** F (22, 440) = 0.1883; **Time** F (2.299, 45.99) = 28.09; **Group** F (1, 20) = 0.07874	**Time x Group**: P>0.9999; **Time**: P<0.0001; **Group**: P=0.7819		
Fig. 4H	**Ctrl** (9cre-, light+; 4 no light[†]) N =13 (6 male, 7 female); **Stim** N = 9 (3 male, 6 female)	RM mixed-effects analysis: Time x Group F (24, 475) = 0.2635; Time F (1.795, 35.52) = 7.957; Group F (1, 20) = 0.07470	**Time x Group**: P=0.9999; **Time**: P=0.0019; **Group**: P=0.7874		
Fig. 4I	**Ctrl** (9cre-, light+; 4 no light[†]) N =13 (6 male, 7 female); **Stim** N = 8 (3 male, 5 female)	Two-way RM ANOVA: Time x Group F (1,20) = 1.380; Time F (1,20) = 30.76; Group F (1, 20) = 0.1755	**Time x Group**: P=0.2539; **Time**:P<0.001; **Group**: P=0.6798	Bonferroni's MCT: 0-5min P=0.975w; 5-10min P=0.5853	1 stim excluded due to video capture issue
Fig. 4J	**Ctrl** (9cre-, light+; 4 no light[†]) N =13 (6 male, 7 female); **Stim** N = 9 (3 male, 6 female)	Two-tailed unpaired t test	P=0.0117		
Fig. 4K	**Ctrl** (9cre-, light+; 4 no light[†]) N =13 (6 male, 7 female); **Stim** N = 9 (3 male, 6 female)	Two-way RM ANOVA: **Time x Group** F (2, 40) = 1.549; **Time** F (1.748, 34.96) = 47.70; **Group** F (1, 20) = 0.02660	**Time x Group**: P=0.2248; **Time**: P<0.0001; **Group**: P=0.8721	Bonferroni's MCT: 2min P=0.1118; 4min P>0.9999; 6min P>0.9999	
Fig. 4L	**Ctrl** (9cre-, light+; 4 no light[†]) N =13 (6 male, 7 female); **Stim** N = 9 (3 male, 6 female)	Two-way RM ANOVA: **Time x Group** F (2, 40) = 1.018; Time F (2, 40) = 8.31; **Group** F (1, 20) = 0.2688	**Time x Group**: P=0.3705; **Time**:P=0.0008; **Group**: P=0.6098	Bonferroni's MCT: 2min P>0.9999; 4min P>0.9999; 6min P=0.7620	
Fig. S1D	2-4 mice per bregma level, 1-2 male, 1-2 female				
Fig. S1F	3 mice, total of 330 neurons counted				
Fig. S1I	2-4 mice per condition, 1-2 male, 1-2 female				
Fig. S1L-S	all images from 1 $Ucn^{cre/+}$ mouse				
Fig. S2F	N = 9 (5 male, 4 female); data points represent the average of 3-15 epochs per mouse	RM one-way ANOVA F (1.056, 8.446) = 6.648	P=0.0302	Tukey's MCT: (-10 to -5) vs (-5 to 0) P= 0.7503; (-10 to -5) vs (0-5) P=0.2843 ; (-10 to -5) vs (5-10) P=0.057 ;(-5 to 0) vs (0 to 5) P=0.3189; (-5 to 0) vs (5-10) P=0.0839; (0 to 5) vs (5-10) P=0.0001	
Fig. S2G	N = 6 (3 male, 3 female); data points represent the average of >20 epochs per mouse	RM one-way ANOVA F (2.511, 12.55) = 30.82	P<0.0001	Bonferroni's MCT: (-10 to -5) vs (-5 to 0) P= 0.1660; (-5 to 0) vs (0 to 5) P =0.0005; (-5 to 0) vs (5-10) P= 0.0023	
Fig. S3E	**Ctrl** (cre-, light+) N =3 (2 male, 1 female); **Stim** N = 4 (3 male, 1 female)	Left: Unpaired Welch's t test and Right: Two-way RM ANOVA: Time x Group F (2,103) = 5.314; Time F (1.312, 6.559) = 81.68; Group F (1, 5) = 22.83	Left: P=0.0003; Right: Time x Group: P=0.0268; Time: P<0.0001; Group: P=0.0050	Bonferroni's MCT: day 1 P=0.0153; day 3 P=0.0352; day 5 P=0.3200	

Fig. S3F	**Ctrl** (cre-, light+) N =8 (5 male, 3 female); **Stim** N = 5 (1 male, 4 female)	Two-way RM ANOVA: **Time x Group** F (3, 33) = 8.935; Time F (3, 33) = 49.61;**Group** F (1, 11) = 16.23	**Time x Group**: P=0.0002; **Time**:P<0.0001;**Group**: P=0.0020	Bonferroni's MCT: 0-5 P>0.9999; 0-15 P=0.0502; 0-30 P=0.0007 0-60 P<0.0001	
Fig. S3G	**Ctrl** (cre-, light+) N =6 (4 male, 2 female); **Stim** N = 5 (1 male, 4 female)	Two-way RM ANOVA: **Time x Group** F (2, 18) = 0.6064; Time F (2, 18) = 83.60;**Group** F (1, 9) = 5.934	**Time x Group**: P=0.5561; **Time**:P<0.0001;**Group**: P=0.0376	Bonferroni's MCT: 0-5 P=0.1988; 0-15 P0.2405; 0-30 P0.0388	
Fig. S3H	**Ctrl** (cre-, light+) N =8 (4 male, 4 female); **Stim** N = 5 (4 male, 1 female)	Left: Two-way RM ANOVA: **Time x Group** F (1, 11) = 3.430; Time F (1, 11) = 83.61;**Group** F (1, 11) = 3.305 ; Right: Simple linear regression Slopes equal F=0.00368. DFn=1, DFd=9; Intercepts Equal F=15.5. DFn=1, DFddddd=10	Left: **Time x Group**: P=0.0910; **Time**:P<0.0001;**Group**: P=0.0964 ; Right: Slopes equal P=0.9530; Intercepts equal P=0.0028	Left: Bonferroni's MCT 0-15 P=0.8818; 0-30 P=0.0446	
Fig. S3I	**Ctrl** (cre-, light+) N =8 (4 male, 4 female); **Stim** N = 5 (4 male, 1 female)	Left: Two-way RM ANOVA: **Time x Group** F (3, 36) = 1.831; Time F (1.606, 19.28) = 4.562;**Group** F (1, 12) = 2.568; Right: Two-way RM ANOVA: Time x Group F (5, 60) = 0.8202; Time F (3.691, 44.30) = 29.33; GroupF (1, 12) = 0.1765	Left: **Time x Group**: P=0.1590;**Time**: P=0.0304;**Group**: P=0.1350; Right: Time x Group: P=0.5401; Time:P<0.0001; Group: P=0.6818	Left: Bonferroni's MCT d4 P>0.999 ; d6 P>0.999 ; d7 P=0.3741 ; test P=0.0987; Right: Bonferroni's MCT all timepoints P>0.9	
Fig. S4B	N = 5 (3 male, 2 female); data points represent 1 epoch per animal for each position	Two-tailed paired t tests	Belly up P=0.4881; sideways P=0.2365; down P=0.9474		
Fig. S4C	N = 3 (2 male, 1 female); data points represent >4 footshocks per animal	Ordinary one-wa y ANOVA: F (2, 6) = 0.1658	P=0.8510	Tukey's MCT: 0.07 vs .15-.2 P= 0.8375; 0.07 vs .3-.4 P= 0.9552; .15-2 vs .3-.4 P=0.9562	
Fig. S4E	N = 9 (5 male, 4 female); data points represent >5 CS epochs and >10 freezing epochs per animal	paired t tests	CS P=0.4479; freezing P=0.0003		
Fig. S5A	**Ctrl** (cre-, light+) N = 4 (3 male, 1 female); **Stim** N = 5 (2 male, 3 female)	Two-way RM ANOVA: **Time x Group** F (2, 18) = 0.6064; Time F (2, 18) = 83.60;**Group** F (1, 9) = 5.934	**Time x Group**: P=0.0528;**Time**: P=0.4462;**Group**: P=0.1352	Bonferroni's MCT: 0-10 P=0.3083; 10-20 P=0.3057; 20-30 P=0.2917; 30-40 P>0.9999	
Fig. S5B	**Ctrl** (cre-, light+) N = 4 (1 male, 3 female); **Stim** N = 8 (4 male, 4 female)	Two-way RM ANOVA: **Time x Group** F (3, 30) = 0.0982; Time F (2.628, 26.28) = 17.44;**Group** F (1, 10) = 1.492	**Time x Group**: P=0.9604; **Time**:P<0.0001;**Group**: P=0.2499	Bonferroni's MCT: OFF 0-2 P>0.9999 ; ON 2-4 P>0.9999; OFF 4-6 P=0.4770 ; ON 6-8 P>0.9999	
Fig. S5C	**Ctrl** (cre-, light+) N =13 (6 male, 7 female); **Stim** N = 9 (3 male, 6 female)	Two-way RM ANOVA: **EPM: Time x Group** F (1,20) = 4.847; Time F (1,20) = 52.45;**Group** F (1, 20) = 2.973. **OFT: Time x Group** F (2,18) = 1.264; **Tim**e F (1.713,15.42) = 10.06;**Group** F (1, 9) = 1.455	**EPM: Time x Group**: P=0.0396; **Time**:P<0.0001;**Group**: P=0.1001. **OFT**: **Time x Group**: P=0.3065;**Time**: P=0.0022 **Group**: P=0.2584.	Bonferroni's MCT **EPM**: 0-5min P=0.0216; 5-10 P>0.9999; OFT: 0 10min P= 0.8048; 10-20min P= 0.2553; 20-30min P= 0.9263	ROUT Q=1% found 1 outlier in the stim group which was excluded from the analysis
Fig. S5D	**Ctrl** (9cre-, light+; 4 no light[†]) N =13 (6 male, 7 female); **Stim** N = 9 (3 male, 6 female)	Left: unpaired t test; Right: Two-way RM ANOVA: Time x Group F (2, 40) = 1.137; Time F (1.97, 39.33) = 76.41; Group F (1, 20) = 0.3643	**Left:** P=0.1192 ; **Right Time x Group**: P=0.3309;**Time**:P<0.0001;**Group**: P=0.5529		

Notes:

† 'no light' controls were attached to patch cables, the laser was simply not turned on

†† sham implant animals underwent the same surgical procedures but had a broken off fiber optic cannula cemented in place.
